# Object-directed action representations are componentially built in parietal cortex

**DOI:** 10.1073/pnas.2421032122

**Published:** 2025-08-20

**Authors:** Leyla Roksan Caglar, Jon Walbrin, Emefa Akwayena, Jorge Almeida, Bradford Z. Mahon

**Affiliations:** ^a^Department of Psychology, Carnegie Mellon University, Pittsburgh, PA 15213; ^b^Faculty of Psychology and Educational Sciences, Proaction Lab & Center for Research in Neuropsychology and Cognitive-Behavioral Interventions (CINECC), University of Coimbra, Coimbra 3000, Portugal; ^c^Neuroscience Institute, Carnegie Mellon University, Pittsburgh, PA 15213; ^d^Department of Neurosurgery, University of Rochester Medical Center, Rochester, NY 14642

**Keywords:** functional MRI, object grasping, tool use, supramarginal gyrus, predictive encoding

## Abstract

The inferior parietal lobule supports action representations that are necessary to grasp and use objects in a functionally appropriate manner [S. H. Johnson-Frey, *Trends Cogn. Sci.* **8**, 71–78 (2004)]. The supramarginal gyrus (SMG) is a structure within the inferior parietal lobule that specifically processes object-directed patterns of manipulation during functional object use. Here, we demonstrate that neural representations of complex object-directed actions in the SMG can be predicted by a linear encoding model that componentially builds complex actions from an empirically defined set of kinematic synergies. Each kinematic synergy represents a unique combination of finger, hand, wrist, and arm postures and movements. Control analyses demonstrate that models based on image-computable similarity (AlexNet, ResNet50, VGG16) robustly predict variance in visual areas, but not in the SMG. We also show that SMG activity is specifically modulated by kinematic (as opposed to visual) properties of object-directed actions. The action-relevant, as opposed to visually relevant, nature of the representations supported by the SMG aligns with findings from neuropsychological studies of upper limb apraxia. These findings support a model in which kinematic synergies are the basic unit of representation, out of which the SMG componentially builds object-directed actions. In combination with other findings [Q. Chen et al., *Cereb. Cortex* **28**, 2162–2174 (2018)], we suggest that kinematic synergies are related to complex object-directed actions in a similar way to how articulatory and voicing features combine to form phonological segments in spoken language production.

## Significance

A central goal of cognitive brain research is to specify the elemental parts out of which complex higher-order representations are constructed, and to articulate how those parts are functionally and neurally implemented. We show that representations of complex-object directed actions in the inferior parietal lobule can be modeled as combinations of finger, hand, wrist, and arm postures and movements—kinematic synergies. These findings motivate a neurocognitive model of how the inferior parietal lobule, in interaction with occipito-temporal and frontal sites, componentially builds complex object-directed actions. In combination with other findings, we suggest that kinematic synergies are combined to form object-directed actions, similarly to how articulatory and voicing features combine to form phonological segments.

Activities of daily living depend on the ability to grasp and use everyday objects according to their function and current action goals. Object-directed actions are supported by dissociable systems that process visual shape and form (1–3), visual surface texture and color (4–7), visuomotor transformations for grasping and manipulation (8–12), and conceptual representations of object function (13–16) (for recent discussion, see ref. 17). Much progress has been made in understanding how visual and visuomotor representations of objects are organized and represented, and how visual representations of objects are processed across ventral and dorsal stream hierarchies (1, 18–25). For instance, face stimuli have been parametrically decomposed into constitutive parts to identify the critical features and their arrangements (eyes, mouth, global shape) that trigger processing in face patches in the ventral stream (26–31). A core idea of componential theories of visual object processing (e.g., refs. 32–34) is that the system productively combines elemental representations in a manner that is robust to variations in viewpoint, occlusion, and noise (e.g., refs. 35–38). This approach is representationally economical and computationally powerful, as a closed set of representations can be productively recombined to form an open set of visual object representations.

Interacting with graspable and manipulable objects involves visual recognition, and the computation of a grasp that is functionally appropriate to how the object will be manipulated in the service of action goals. The brain areas and networks supporting those computations include the supramarginal gyrus, the premotor cortex (precentral gyrus), superior parietal and dorsal occipital regions, the posterior middle temporal gyrus, and the bilateral medial fusiform gyrus along the collateral sulcus (11, 15, 39–56). While those areas have some degree of bilateral representation, there is a bias toward left lateralization for the supramarginal gyrus, premotor cortex, and posterior middle temporal gyrus. That network of regions is consistently engaged when healthy participants view or name manipulable objects during functional MRI (fMRI) compared to a range of baseline categories (faces, places, animals, and printed words).

A long history of cognitive neuropsychological research in individuals with focal lesions has identified the parietal lobule as a key substrate for object-directed action. The anterior intraparietal sulcus supports visuomotor transformations for hand shaping in the service of object grasping (43, 46), while the left supramarginal gyrus supports praxis representations, or complex object manipulations associated with functional goal-directed object use (15, 39, 52, 56–66). Functional neuroimaging has provided support for the hypothesis that the left supramarginal gyrus supports access to representations of complex object-directed manipulation. This has been demonstrated using a range of experimental paradigms, including overall univariate amplitude of the BOLD response, repetition priming as a function of action and object repetition, and multivariate decoding of patterns of object directed action (9, 15, 67, 68). Despite knowing that the supramarginal gyrus supports complex object manipulation, *how* this structure represents and generates representations of object-directed actions is unknown.

Here, we test the hypotheses that a) object-directed action representations are componentially built from kinematic synergies, and that b) the left supramarginal gyrus is the principal region supporting that componential process. In this context, kinematic synergies are independently defined postures and movements of the finger, hand, wrist, and arm that encompass all possible hand movements (69).

## Testing a Componential Model of Object-Directed Actions: Primary Hypothesis

The core hypothesis tested in the current investigation is that kinematic synergies form the building blocks out of which complex object-directed action representations are built, and that this process is supported by the supramarginal gyrus. Prior empirical work in neuroprosthetics and robotics (70–73) has identified feature covariations that form hand postures or synergies for grasping and manipulating objects. Our investigation started by stipulating a closed set of kinematic synergies that were derived from prior studies. Starting with a fixed set of kinematic synergies can be contrasted with the strategy of using computational or data-reduction approaches to derive a parcellation of features that best explains the neural data for a given set of objects (16, 74–76). The approach of starting with a closed set of kinematic synergies has the advantage that the model’s generative nature makes it extensible to an open-ended set of objects. What is meant here by generative is that an open set of object-associated action representations can be built through recombinations of a closed set of kinematic synergies. We thus use kinematic synergies, as previously defined in the literature, as a fixed set of features within an encoding framework to predict neural responses in the supramarginal gyrus.

There is direct precedent to the idea that action representations are built from kinematic synergies. Prior work has emphasized a network of areas including the bilateral precentral cortex, supplementary motor cortex, and inferior parietal regions as collectively encoding kinematic synergies of the hand (77). Prior work has also advanced the basic computational idea that a generative process supports the combination of finger movements into hand movements to create more complex actions (78, 79). That prior literature did not consider the role of kinematic synergies in generating actions toward objects, nor the role of the inferior parietal lobule as a site that might support the componential production process for transitive actions. Advancing these issues with respect to real-world familiar object representations and their associated actions addresses a key gap in our understanding of how to model the neural processes that give rise to common activities of daily living. It is important to clarify in this regard that we are not studying motor control, and participants in the studies reported herein are not performing overt actions. Rather, we study the brain’s action systems by using images of manipulable objects as visual stimuli that automatically engage parietal action systems. This is based on the long-standing observation (15, 44, 45) that viewing manipulable objects, with no explicit intention to use them, leads to the automatic evocation of action-relevant representations in the left supramarginal gyrus, among other regions. We are thus interested in studying object-directed action representations, which incorporate not just motor-relevant processing, but also perceptual, conceptual, and anticipatory proprioceptive and somatosensory processing (see additional considerations in *Discussion*).

Our analytic approach builds on prior investigations into the componentiality of noun representations. In a now classic demonstration, Mitchell and colleagues (80) used an encoding model to predict neural activations associated with the meaning of nouns via a weighted linear sum of contributions from each of the word’s associated semantic features. That model was able to predict a noun’s (e.g., “celery”) brain activation based on the patterns of activation of constituent features (e.g., “eat,” “taste,” and “full”), each weighted by a corpus statistic of how strongly the features were associated with the to-be-predicted noun. Here, we develop an analogous empirical approach to isolate whole-brain neural maps for each feature (kinematic synergy). We test whether a linear encoding model that generates a weighted combination of kinematic synergy brain maps can componentially “build” neural representations of object-directed actions. A successful demonstration would consist of showing that an object’s action-related brain activity can be estimated from combinations of kinematic synergies, where the relevance of each synergy to each object is set empirically by independent ratings. We test that prediction using a linear kinematic encoding model to predict fMRI activity in the supramarginal gyrus, as an a priori defined region in which to test the core hypothesis.

## Testing Underlying Representational Form and Content: Secondary Hypotheses

Objects can share similarities along various action-relevant dimensions (16, 67, 75, 81). For example, a screwdriver and a corkscrew have little functional similarity, but are manipulated with a similar twisting motion. By contrast, a corkscrew and bottle opener have similar functions, but are manipulated in quite different ways (see ref. 8 for original neuropsychological evidence). Some of those action-relevant dimensions of similarity can be related to dimensions of visual or structural similarity. This is because manipulable objects are designed to have a specific structure that affords certain actions in support of an intended function. This fact about the structure of the world means there is a correlation (in the input statistics) between the visual structure of objects (reflected in image-based statistics of visual or structural similarity) and the kinematics of those objects’ associated actions.

Objects that are grasped and manipulated in similar ways *tend* to have similar structures. Importantly, however, the strength of that relation varies across graspable objects, and it is possible to separate kinematic from visual similarity. The degree to which object function constrains manner of manipulation and functional grasping can be captured by “Centrality of Manipulation to Function” (see data and discussion in ref. 15; for a similar measure, see ref. 82). For manipulable objects for which the associated action is highly central to the function of the object, there is one way (or a very limited number of ways) an object can be manipulated while accomplishing its function. Manipulable objects with high centrality of manipulation to function are referred to as “tools.” Note the term tools does not pick out a strict category, but rather an empirically defined subclass of graspable objects for which there is a tight relation between structure, function, and manner of manipulation (83). Examples of tools would be “hammer,” “toothbrush,” or “fork.” By contrast, other equally graspable and familiar objects, have a comparatively unconstrained relation between function and manner of manipulation. For instance, one can hold a book in myriad ways and turn the pages with different actions and still accomplish reading. Graspable objects with low centrality of manipulation have been referred to as “arbitrarily manipulated objects,” to capture the intuition that they can be grasped and manipulated in varied or arbitrary ways in different contexts, while still achieving the function of use of the object (15).

Because the goal of the current project is to test whether kinematic synergies are combined to form complex actions, it is important to use stimuli with high Centrality of Manipulation. This is because, for objects with high Centrality of Manipulation, the same kinematic synergies will tend to be activated across different perceptual interactions with the object. This is ideal for testing the primary hypothesis of whether object-directed action representations in parietal cortex are built from an empirically defined closed set of kinematic synergies. These considerations also drive two secondary hypotheses. First, an encoding model based on kinematic features that successfully predicts neural variance (i.e., fMRI BOLD signal) in parietal cortex would also be expected to explain variance in visual regions of the ventral and dorsal pathways that represent object structure. On the other hand, however, success of the kinematic encoding model in explaining variance in the supramarginal gyrus might be attributed to visual/structural (rather than kinematic) dimensions. We thus conduct a series of whole-brain and ROI-based head-to-head tests of the kinematic encoding model versus three image-based deep neural networks (AlexNet, VGG16, ResNet50), as well as a representational dissimilarity model derived from participant judgments of objects’ visual features. To anticipate our findings, while the kinematic and visual models all predict variance in visual regions of the brain, the kinematic model predicts significantly more neural variance than the visual control models in the supramarginal gyrus. Second, we sought positive evidence that neural representations in the supramarginal gyrus are driven by kinematic processing, as opposed to visual processing. We find that ratings of Centrality of Manipulation to function positively predict the amplitude of neural responses in the supramarginal gyrus across objects, indicating the kinematic (as opposed to visual) nature of the representations supported by the supramarginal gyrus. Finally, we report a full replication of our core finding, with a new group of fMRI participants and set of objects intentionally selected to decorrelate action from visual similarity.

## Results

### An Action Similarity Space for Objects Based on Kinematic Synergies.

**Fig. 1.**
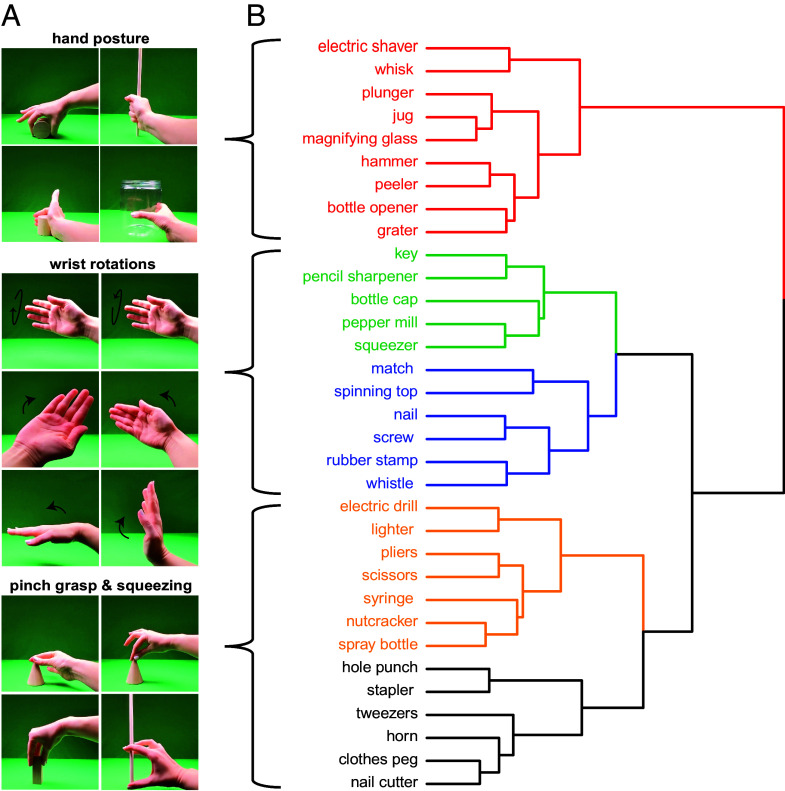
Object similarity computed from kinematic synergies. (*A*) Example frames from videos depicting kinematic synergies. Each kinematic synergy captures a combination of hand posture, joint angles, and finger, wrist, forearm, and shoulder movements. All possible manual actions can be expressed as combinations of kinematic synergies. (*B*) Dendrogram of object similarity based on kinematic synergies demonstrates meaningful clustering. Each object was projected as a weighted vector in the 54-dimensional state space defined by the closed set of kinematic synergies. The dendrogram serves to visualize the validity of this approach for capturing relevant similarity among the objects in terms of their associated actions. Subclusters can be identified as having shared features of hand shape postures (red), manipulations that involve a wrist rotation (blue and green), and squeezing motions that incorporate a palmar or nonpalmar pinch grasp (yellow and black).

We developed a set of 54 kinematic synergies by aggregating synergies across prior independent studies (70–73) and eliminating redundancies when possible (Fig. 1*A* for example kinematic synergies). Each of the 54 kinematic synergies was recorded as a short video (Materials available via our repository). In parallel, we selected a large set of common objects representing tools, arbitrarily manipulated objects, and some large nonmanipulable objects. Naïve participants (n = 30; see Experiment 3 and *SI Appendix* for details) judged, for each of the objects, the “degree to which the pattern of motor movement associated with the object is central to the object’s function.” That judgment captures “Centrality of Manipulation.” Based on those ratings, we focus core hypothesis testing on the subset of objects (n = 33) for which the pattern of movement was judged to be highly central to the object’s function. This was determined empirically, with an independent rating experiment (based on a median split of objects based on centrality ratings, *SI Appendix*, Fig. S1).

A separate group of naïve participants (n = 87) judged whether each of the 54 kinematic synergies is involved in grasping or manipulation of each of the selected objects (see *Methods*, Experiment 2, and *SI Appendix* for details). The resulting matrix of kinematic synergies by objects was used to compute the similarity among the objects in terms of their associated kinematic synergies. Each of the 33 objects could be projected into a 54-dimensional kinematic state space. The dendrogram in Fig. 1*B* represents a visualization of the similarity among the objects based on kinematic synergies. The objects cluster into intuitive groups based on the similarity of their associated actions. We compared those results to a behavioral similarity dataset published by Almeida and colleagues (16). Almeida and colleagues measured similarity among the same object stimuli with a classic “piling task,” in which participants were asked to group objects based on their intuitions of how those objects are manipulated. We used entanglement to measure the alignment between the dendrogram generated with our approach of calculating similarity “bottom–up” using kinematic synergies, with the dendrogram generated with the approach using the behavioral piling task (*SI Appendix*, Fig. S2). The results of this comparison demonstrated high alignment (entanglement value = 0.12) between the two dendrograms’ clusters. Entanglement quantifies the similarity in hierarchical clustering trees. Entanglement values range from 0 (perfectly aligned) to 1.0 (completely different). A permutation test of randomly shuffled dendrograms (10,000 permutations) showed that the value of 0.12 lies well outside the distribution of entanglement values that are expected by chance. Overall, this analysis shows that the bottom–up approach of computing object similarity based on kinematic synergies is in good agreement with more traditional approaches of asking participants to rate the action similarity among the objects.

### Kinematic Similarity Explains Neural Representational Similarity.

The empirical goal of this study is to test whether neural responses in the left supramarginal gyrus are predicted by an encoding model that represents only kinematic synergies. As a first step, we tested whether object similarity, as captured by kinematic synergies, explains neural patterns in the relevant parietal region using classic multivoxel representational similarity analysis (RSA). Participants (N = 25) completed a categorization task (animal/tool) during fMRI over the object stimuli used in the behavioral norming studies (see *Methods* for details, Experiment 1). Importantly, the task completed during fMRI did not involve any consideration of object-directed actions, nor engage participants in any explicit action intentionality. This was by design, because we are interested in the action-relevant information that is automatically computed about visually presented manipulable objects. A searchlight RSA (74, 84) was used to compute a whole-brain map of where kinematic state space similarity predicted neural patterns. At each searchlight, a representational similarity matrix (RSM) of the neural similarity among the 33 objects was computed and compared to the RSM based on kinematic synergies (i.e., the 54-dimensional kinematic state space, see *Methods*). The searchlight analysis was carried out in each subject’s native space and corrected via Threshold Free Cluster Enhancement at the group level [TFCE (85); 10,000 permutations; see *SI Appendix* for details].

**Fig. 2.**
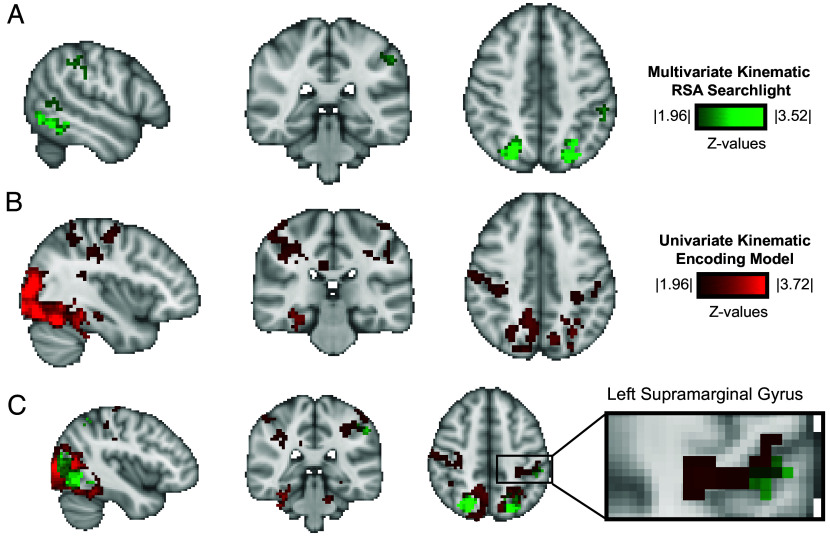
Neural responses in the inferior parietal lobule predicted by object-associated kinematic synergies. (*A*) A whole-brain multivariate searchlight RSA identified the left supramarginal gyrus, among other regions known to support object recognition and object-directed action (*P* < 0.05 TFCE corrected). (*B*) The whole-brain univariate encoding model predicts neural responses in the supramarginal gyrus, and other areas of parietal cortex (*P* < 0.05 TFCE corrected). This model identifies voxels for which there is significant prediction of out-of-set (i.e., untrained) objects, based on the projection of those objects in the 54-dimensional state space defined by kinematic synergies. (*C*) Overlap between the classic multivariate RSA searchlight and the univariate kinematic encoding model identifies voxels in the left supramarginal gyrus (*P* < 0.05 TFCE corrected; MNI coordinates: −39,−34,47).

If the kinematic synergies capture relevant representational similarity, the left supramarginal gyrus should be identified via a whole-brain search. We also expected, due to the intrinsic relation between object structure and object-directed action, that the kinematic similarity space would explain variance in neural similarity within visual regions of the ventral and dorsal pathways that code visual attributes of objects. Confirming both predictions, the resulting map identified the left supramarginal gyrus (Fig. 2*A* and Table 1 for coordinates, *SI Appendix*, Fig. S3 for slices covering the whole brain) as well as other areas known to support object-directed action and object recognition, including anterior intraparietal sulcus (aIPS) bilaterally, the premotor cortex, and somatosensory regions. Also as anticipated, the multivariate RSA searchlight analysis identified visual regions of the dorsal and ventral pathways, including the bilateral dorsal occipital cortex, and ventral and lateral temporal-occipital regions. These initial observations validate the kinematic state space as a means with which to study the representational structure of how the brain represents object-directed actions. Subsequent analyses sought to gain insight into the underlying componential mechanisms and to dissociate kinematic from visual dimensions.

**Table 1.** Peak voxels and cluster sizes for Experiment 1: The Multivariate Kinematic RSA Searchlight, Experiment 1: Univariate Kinematic Encoding Model, and Experiment 4: Univariate Kinematic Encoding Model

R	Exp 1: Multivariate Kinematic RSA Searchlight					Exp 1: Univariate Kinematic Encoding Model					Exp 4: Univariate Kinematic Encoding Model				
ROIs	L/R	MNI			Size	L/R	MNI			Size	L/R	TAL			Size
SMG	L	−54	−34	44	47	L	−54	−37	56	52	L	−55	−23	34	98
						R	45	−22	38	468					
aIPS	R	24	−55	53	17	L	−30	−34	44	130	L	−30	−31	39	122
SPL	R	16	−59	63	8	L	−36	−61	47	2	L	−18	−66	52	199
											R	17	−65	50	39
Fusiform gyrus	R	30	−55	−13	29	L	−60	−49	11	65	R	43	−57	−6	56
	L	−25	−55	−18	24						L	−41	−53	−12	55

### Object-Directed Action Representations Modeled As Linear Combinations of Kinematic Synergies.

To test whether object-directed action representations in the supramarginal gyrus can be modeled as linear combinations of kinematic synergies, we tested out-of-set prediction of neural data for untrained objects. The encoding model predicts univariate voxel activity, for an object that was left out of the model’s dataset, from a linear weighted combination of the whole-brain kinematic synergy maps. The weighting is based on independent ratings of the degree to which each kinematic synergy is involved in the grasping and manipulation of each object.

For each subject, the predictive encoding model was constructed in two steps (*SI Appendix*, Fig. S4). In the first step, the goal was to compute a whole brain map for each synergy. To do this, the to-be-predicted object was left out, starting at the first step, such that the entire process described here was iterated across the 33 objects (at each voxel, for each participant). A whole-brain fMRI activation map was computed for each of the 33 object stimuli. Based on behavioral judgments (Experiment 2, *Methods*), each synergy had a rating ranging from 0 to 1 (averaged across subjects), for each object. That rating corresponded to the proportion of subjects who rated that synergy as being relevant for that object. We determined, for each kinematic synergy (s), the subsets of objects that were ranked high (greater than 0.75) and low (less than 0.25) for that synergy, always excluding the to-be-predicted object (t). This allowed us to compute, in a purely data driven manner, a whole-brain contrast to identify patterns of neural findings (across objects) that corresponded to high (vs. low) relevance for a given synergy. Note that this step, of defining the objects that were high and low for synergy (s) was independent of how the other 53 synergies may have ranked. A univariate contrast subtracted the whole-brain BOLD contrast map of each object rated low on synergy *s* from the contrast map for each object rated high for synergy *s.* The resulting set of univariate contrast maps were averaged, to create a whole brain map that represents synergy *s*. This was repeated for each of the 54 synergies, resulting, for each kinematic synergy, in a whole brain kinematic synergy map. As described above, these maps were obtained always excluding the to-be-predicted object and generated at the individual subject level. Those whole-brain synergy maps served as the basis for building the predictive encoding model.

$$y_{v}=\sum_{i=1}^{n}s_{\mathrm{vi}}k_{i}(t).$$

#### Formula 1: Predicting an object’s activity at each voxel (y_v_) based on a linear combination of kinematic synergies (s_vi_), weighted by their rated relevance (k_i_) to that object (t), over all kinematic features (n).

In the second step, each to-be-predicted object’s neural activation (t) was predicted at every voxel (y_v_) as the linear combination of the object’s kinematic synergy maps (s_vi_). The contribution of each whole-brain synergy map to the eventual prediction is weighted by an empirical measure (Experiment 2 ratings) of the relevance of that synergy (k_i_) for that object. This was iterated over all kinematic features (n) (see Formula 1, and *SI Appendix*, Fig. S5 for a visual schematic). We repeated this two-step approach for each object (i.e., 33 folds, each time going back to Step 1, so that the to-be-predicted object was withheld from the trained model). The result is that the predicted whole-brain activation map for each object is based on synergy maps that were computed independently of that target object.

Because the encoding model is a univariate model, and because the model predicts each object’s representation, rather than the similarity structure among objects, we refer to this as a *univariate RSA*. The encoding model generates, for each subject (N = 25), and each voxel, a vector of all objects’ predicted BOLD activity. We then correlated, within each voxel, and within each subject, the model’s predicted pattern of responses for the 33 objects with the observed pattern of responses to those 33 objects. The resulting correlation coefficients were Fisher transformed and compared to zero at the group level with a two-tailed, one-sample, *t* test. Group t-maps were corrected using TFCE over all subjects (*P* < 0.05; see *Methods*). This resulted in a single whole-brain t-map representing each voxel’s prediction accuracy (across all subjects) for out-of-set objects, based on an encoding model that represents only object-associated kinematic synergies and their relative importance for each object. The primary test is whether this model identifies, in a whole-brain random-effects analysis, the left supramarginal gyrus. As noted (see Secondary Hypothesis Testing), the model is anticipated to also explain variance in visually responsive brain regions, due to the relation between kinematic synergies and object structure.

### Primary Hypothesis Testing: Univariate Kinematic Encoding Model.

In line with the primary prediction, the univariate encoding model predicted neural responses to objects in the inferior parietal lobule, including the left supramarginal gyrus centered around PFm, as well as the right supramarginal gyrus centered around PFt (Fig. 2*B* and Table 1), and bilaterally in PFop. Grasp-relevant areas were also identified, including bilateral superior parietal, left precuneus, dorsal occipital cortex [V6/V6a (86, 87)]. The model also predicted responses in the left hemisphere primary hand motor cortex and the right cerebellum, the latter of which is known to support motor coordination and have an ipsilateral organization (16, 88–90). There was overlap (Fig. 2*C*) between the voxels of the left supramarginal gyrus identified by the univariate kinematic encoding model and those identified by the multivariate kinematic RSA searchlight. The focus of overlap in the supramarginal gyrus also shows excellent overlap with previous findings in the literature identifying tool-relevant areas and using Centrality of Manipulation to define tool stimuli (9, 15, 40, 45, 67, 81, 91) (see *SI Appendix*, Table S1 for peak voxel coordinates from the literature). Furthermore, and as anticipated, the kinematic encoding model identified bilateral ventral and lateral occipital-temporal regions (see *SI Appendix*, Fig. S6 for full brain coverage in coronal slices).

### Secondary Hypothesis Testing: Separating Kinematic from Visual/Structural Similarity.

The tight mapping between object-directed actions and object structure motivated the expectation that an encoding model that represents kinematic synergies will predict neural variance *both* in the supramarginal gyrus as well as in ventral and dorsal stream *visual* regions. However, it might be argued that the responses in the critical test region, the supramarginal gyrus, could also be explained by visual/structural dimensions rather than by kinematic dimensions. Indeed, parietal cortex is visually responsive and processes visual representations that generalize in important ways beyond the input image (1, 25, 46, 49, 51, 92–94). We conducted two control analyses, corresponding to secondary hypotheses as described in the Introduction, to test whether neural responses in the supramarginal gyrus are driven by kinematic or visual dimensions. The first approach shows that models based on image computable information, and on human behavioral judgments of visual similarity, do not account for neural responses in the supramarginal gyrus but do predict responses throughout the ventral and dorsal visual pathways. The second approach demonstrates positive evidence that kinematic dimensions, rather than vision-based dimensions, modulate responses in the supramarginal gyrus.

#### Whole-brain analyses.

We first tested whether the supramarginal gyrus identified by the kinematic encoding model is also identified by models that represent objects only in terms of image computable properties. To that end, we used four control visual models. The first model is based on published data of behaviorally judged visual similarities from an object piling task [*SI Appendix*; (16)]. The other three models were pretrained convolutional neural networks (CNNs) with different architectures: AlexNet (95), ResNet50 (96), and VGG16 (97). For each of the four visual models, a predictor RSM of model-derived interobject similarity was used to predict neural variance in a whole-brain RSA searchlight analysis (*Methods*). For the CNNs, we extracted the objects’ representational similarity, based on the network’s neuronal weights associated with each object in the first maxpool layer, to generate the predictor RSM. Group-level results were computed using two-tailed one-sample *t* tests (after Fisher transformation) and corrected for multiple comparisons with TFCE (10 k permutations, at *P* < 0.05).

**Fig. 3.**
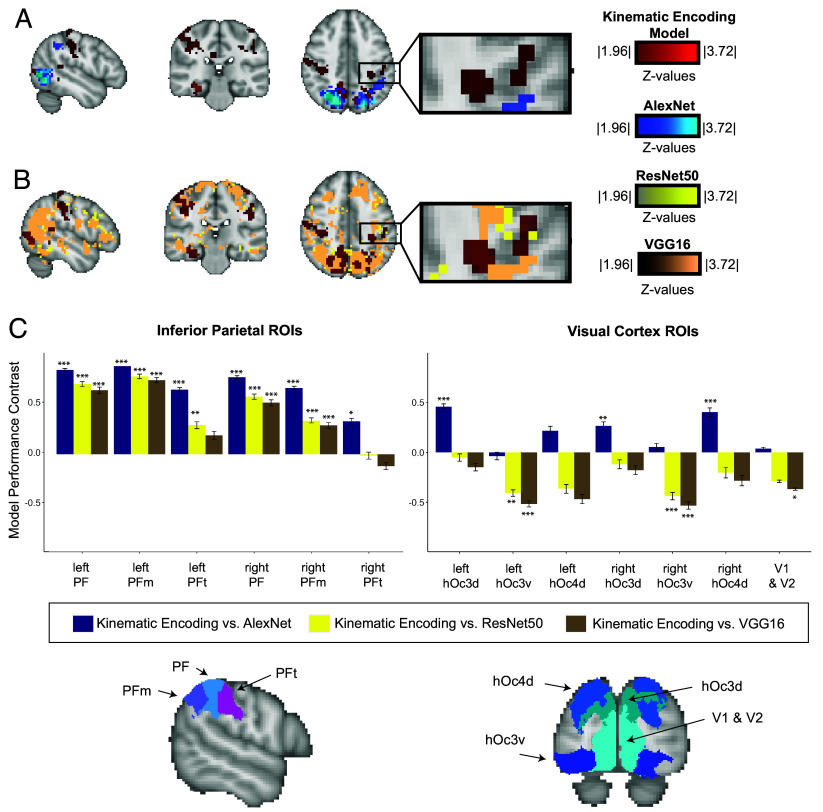
Control analyses rule out that the supramarginal gyrus codes visual dimensions of object representation. (*A*) The CNN model AlexNet, which represents only visual properties of object similarity, does not predict neural responses in the supramarginal gyrus (*P* < 0.05 TFCE corrected). The regions of the supramarginal gyrus identified by the kinematic encoding model are shown for comparison, and do not overlap with those identified by AlexNet. (*B*) The CNN models ResNet 50 and VGG16 do not predict neural responses in the supramarginal gyrus (*P* < 0.05 TFCE corrected). The results of the kinematic encoding model are overlaid for comparison. (*C*) A region of interest (ROI) analysis shows that the kinematic encoding model explains significantly more variance than the control visual models in inferior parietal ROIs, while the visual control models explain more variance than the kinematic encoding model in visual cortex ROIs. We computed “Model Performance Contrast” as ([Model 1 − Model 2]/[Model 1 + Model 2]). Inferior parietal ROIs (bilateral regions PF, PFm, and PFt) and visual cortex ROIs (bilateral V1&V2, hOc3d, hOc3v, and hOc4d) are based on the JuBrain SPM Anatomy Toolbox [Version 3.0; (99–101) and shown below (x-axis). All plotted data present Model Performance Contrasts based on the r^2^ of the kinematic encoding model vs. each control model (y-axis). A permutation based ANOVA showed a significant interaction between models and ROIs (F(38,936) = 35.3; *P* < 0.001). ROI significance is based on Bonferroni adjusted *P*-values of planned one-sample *t* tests of each comparison against zero; error bars represent the SEM over subjects (N = 25).

**Fig. 4.**
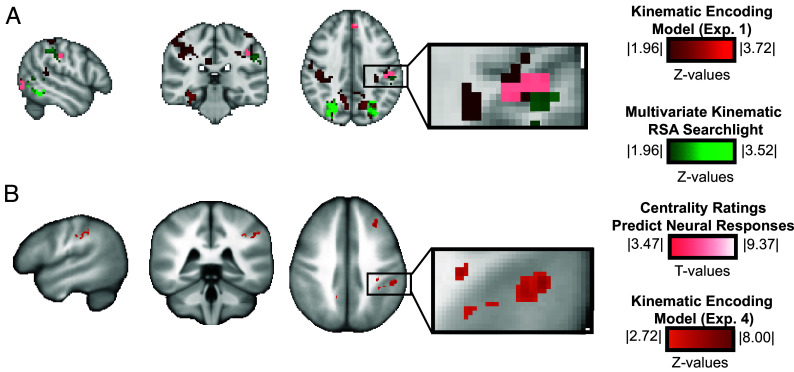
Positive evidence that representational content in the left supramarginal gyrus is kinematic. (*A*) The centrality of objects’ manipulation to their function (Experiment 3) predicts the amplitude of neural responses in the left supramarginal gyrus (*P* < 0.05 FDR corrected; MNI coordinates: −53,−29,38). Those voxels overlap with voxels independently identified by the kinematic encoding model and the Kinematic RSA model. (*B*) Results of Experiment 4, showing a full replication of the core finding that the univariate kinematic encoding model predicts neural responses in the supramarginal gyrus for out-of-set objects (*P* < 0.05 cluster-level corrected, Monte Carlo simulations < 1,000; Talairach coordinates: −49,−34,38).

As expected, the visual model based on behavioral judgments (piling task) predicted neural responses in the bilateral lateral occipital cortex (LOC; see *SI Appendix*, Fig. S7), a region that supports high-level visual shape and form processing (2, 98). Despite overlap in LOC, there was no overlap in any parietal areas, arguing against the role of high-level visual dimensions in accounting for the neural response patterns in the supramarginal gyrus. Furthermore, all three CNN models based on image-computable (i.e., visual) properties of objects identified bilateral regions along the ventral and dorsal visual pathways (Fig. 4 *A* and *B*; see *SI Appendix*, Figs. S8–S10 for all coronal slices). Those visually responsive regions overlap with the regions identified by the kinematic encoding model (Fig. 3 *A* and *B*). Importantly, while visual-based CNN models predicted neural responses in visually responsive posterior parietal regions, no control model predicted neural responses in the left supramarginal gyrus.

#### ROI analyses.

We conducted head-to-head tests of the kinematic encoding model versus the visual models in a series of ROI analyses over ventral and dorsal visual regions (bilateral V1 and V2, and hOc3d, hOc3v, hOc4d) and inferior parietal areas (right and left PF, PFm, PFt; see Table 1). An ROI (13 levels) by Model (6 levels) ANOVA demonstrated a significant interaction (F_(38, 936)_ = 35.3; *P* < 0.001; see *SI Appendix* for model details). The kinematic encoding model significantly outperformed all vision models in bilateral PF and PFm and significantly outperformed AlexNet in bilateral PFt and ResNet50 in left PFt with large group-level effect sizes based on Cohen’s d (see Fig. 3*C*; Bonferroni correction adjusted *P*-values are based on planned one-sample *t* tests, against zero, of the kinematic encoding model’s performance compared to the respective control visual model). All significance values and effect sizes can be found in *SI Appendix*, Table S2. This underlines that inferior parietal neural responses are best explained by the kinematic encoding model, compared to each of the four tested visual control models. On the other hand, the visual models outperform the kinematic encoding model in dorsal and ventral visual regions. These ROI analyses thus recapitulate the pattern that emerged from the whole-brain analyses.

### Positive Evidence for Kinematic Coding in the Supramarginal Gyrus.

As an independent test of the nature of the underlying neural representation in the supramarginal gyrus, we sought positive evidence that responses in this structure track kinematic aspects of object-directed actions. As noted above, core hypothesis testing was performed on objects (n = 33) for which the manipulation was judged to be differentially central to the function of the object (based on the results of Experiment 3; *SI Appendix*, Fig. S1 shows the distribution over 66 objects of the collected centrality ratings). FMRI data were available, however, for the full set of objects (n = 66; Experiment 1) with a wider range of Centrality of Manipulation ratings. If the supramarginal gyrus represents kinematic properties of object-associated actions, then the overall amplitude of neural responses in that region should be parametrically modulated, across items, by Centrality of Manipulation. A whole brain univariate analysis tested the relation between fMRI responses across the full set of objects and normalized centrality ratings. Thus, at each voxel in each subject, the correlation was computed between Centrality of Manipulation and the amplitude of neural responses, across the full set of objects. The resulting single-subject r-maps were Fisher transformed and tested for significance at the group level with a two-tailed one-sample *t* test, correcting for multiple comparisons using FDR cluster correction (*P* < 0.05, cluster-forming height threshold < 0.001). This analysis demonstrated that the amplitude of neural responses in the left supramarginal gyrus were positively related to Centrality of Manipulation, and that this was significant in a random-effects group level analysis across the full stimulus set (Fig. 4*A*; *SI Appendix*, Fig. S11; MNI peak voxels (x,y,z) = −45,−25,38).

One concern that may be raised is whether Centrality of Manipulation is related to familiarity of interaction, and it is rather familiarity of interaction that modulates the amplitude of responses in the supramarginal gyrus. We were able to rule out this possibility because in the same study in which we collected centrality ratings, we also collected familiarity ratings (*Methods*). We thus tested, with a univariate whole-brain analysis, whether familiarity ratings of object interactions predict neural responses in the supramarginal gyrus (*Methods* and *SI Appendix*, Fig. S12). This analysis revealed that while familiarity of manipulation predicted the amplitude of neural responses in bilateral posterior ventral and dorsal visual regions, it did not modulate responses in the supramarginal gyrus (*SI Appendix*, Fig. S12). These findings reinforce the conclusion that the supramarginal gyrus, particularly on the dominant side, supports kinematic properties of object-directed action representations.

### Interim Summary.

Three core findings have been described. First, neural responses in the supramarginal gyrus are predicted by an encoding model that represents object-directed actions through a linear combination of a closed set of kinematic synergies, but not by models that represent objects in terms of high-level visual, or image-computable properties. Second, both kinematic and vision-based models predict neural responses in visual areas of the ventral and dorsal pathways. Third, neural responses in the left supramarginal gyrus are specifically modulated by the Centrality of Manipulation to object function. As a final test of the robustness of the core finding we conducted a full replication experiment, with a new group of participants, to retest the Primary Hypothesis.

### Replication of Primary Hypothesis.

We performed a full replication using a new group of participants (n = 12). Based on what was learned from the results reported to this point, we made three design changes to the fMRI study to improve the experimental design for the study question and goal. First, we selected a subgroup of nine objects that were a) sparsely distributed in kinematic space, b) had high Centrality of Manipulation ratings, and for which we sought (as much as possible) to c) decorrelate kinematic and visual dimensions of similarity (*Methods* and *SI Appendix*, Fig. S1). Second, we slowed down the design to allow the BOLD response to resolve between trials, thus increasing the signal-to-noise on estimates of the neural patterns corresponding to each individual object. And third, we changed the task to a judgment about the center of mass of each object. This was to ensure participants were performing a cognitively absorbing task focused on the objects’ perceptual properties. Processing of the data and kinematic model training and testing were the same, generating group-level whole-brain univariate RSA maps. As shown in Fig. 4*B*, the results represent a direct replication, with a random-effects analysis, of the core finding in the supramarginal gyrus. The higher signal-to-noise introduced by the updated design identifies specifically the *left* supramarginal gyrus, with less involvement of ventral and dorsal visual regions (see *SI Appendix*, Table S3 for measures of model explained variance). Overall, the excellent alignment between our original findings and this independent replication underlines the robustness of the core finding: the left supramarginal gyrus supports object-directed action representations that are well modeled as linear combinations of a closed set of kinematic synergies.

## Discussion

We have tested the hypothesis that the supramarginal gyrus supports the recombination of a closed set of kinematic synergies into object-directed action representations. We found that a linear encoding model that represents only kinematic synergies predicts neural responses in the supramarginal gyrus. Furthermore, while the kinematic encoding model as well as visual models predicted variance in visual regions of the brain, only the kinematic model predicted neural responses in the supramarginal gyrus. As an independent test of the premise that the left supramarginal gyrus codes *kinematic* aspects of object-directed action representations, we showed that the centrality of an object’s manipulation to its function explains variance in the amplitude of responses in the left supramarginal gyrus. Finally, a full replication with a new group of participants showed, again, that the kinematic encoding model predicts neural responses to objects, in random-effects analyses, and specifically in the supramarginal gyrus. These findings support the inference that the supramarginal gyrus contributes to a componential process of building object-directed actions from kinematic synergies.

### Componential Processes Supporting Hand and Mouth Actions.

An important analogue to our investigation of the componentiality of object-directed actions is prior work on the componentiality of speech motor representations. Indeed, one of the original contexts in which componentiality was proposed was in support of the complex actions performed by the lips, tongue, and larynx in the setting of speech motor production (e.g., refs. 102–105). Recent work (106–108) has capitalized on the componentiality of the speech motor system to develop neural-prostheses that map speech motor brain activity to speech sounds, allowing patients in a locked-in state to communicate (see also refs. 109 and 110). That research used the fixed English phono-articulatory feature space to reduce the dimensionality of the learning problem that a neural network must solve when mapping intracranial neurophysiological brain data to words.

A hypothesis that is framed by our findings, in coordination with prior research, is that the supramarginal gyrus supports a similar computation in language and manual action production. The supramarginal gyrus is involved in phonological processing, and particularly in articulatory sequencing (111–114). Lesions to the supramarginal gyrus and surrounding structures, in addition to being associated with limb apraxia, are also associated with phonological processing deficits. Those findings raise the intriguing possibility that the supramarginal gyrus supports a superordinate componential process of relating actions to their constituent parts, both for the hand (functional object grasps to kinematic synergies) and the mouth (phonological segments to phono-articulatory features).

Our methodological approach started with an empirically defined closed set of kinematic synergies and tested whether those features can be productively recombined to form complex action representations. Specifically, we empirically isolated whole-brain feature maps for kinematic synergies via univariate contrasts. This approach has the benefit of interpretability and generalization to a variety of other fMRI datasets and processing domains. Future work may test whether this approach to isolating neural feature maps holds promise for application to other domains where complex representations may be componentially built (e.g., shape, color, speech sounds). For instance, it would be interesting to test whether the same methodological approach can isolate neural variance for phonological articulatory features, that can be recombined to predict neural responses to phonological segments.

This investigation has been theoretically focused on testing a model of supramarginal gyrus function. At the same time, we note that neural representations of kinematic synergies reach far outside of the supramarginal gyrus and depend on processing distributed across ventral and lateral temporo-occipital regions, superior parietal, frontal regions, and subcortical structures. Our argument has been that supramarginal gyrus plays a particularly important role in relating high-level representations of actions to their component parts. Evidence that the supramarginal gyrus represents object-directed actions is provided by prior work which has used a multivariate decoding approach to show that left supramarginal gyrus representations of object-directed actions transfer across objects (67). In that prior work, participants were engaged in pantomiming actions during fMRI scanning. A linear decoding model was trained to distinguish the neural representation of (for instance) “corkscrew” (twisting motion) from “scissors” (squeezing motion) and then tested for transfer to other object pairs: for instance, “screwdriver” (also a twisting motion) vs. “pliers” (squeezing motion). Object-directed action representations in the supramarginal gyrus were found to transfer across different objects that share similar manners of manipulation. The current proposal, that object use representations can be componentially reduced to kinematic synergies, offers a mechanism with which to understand how representations of object-directed action generalize across objects.

### Reciprocal Interactions among Parietal and Temporo-Occipital Areas.

As emphasized already in early discussions of the two visual stream hypothesis (e.g., ref. 115), functional object use requires interactions between the dorsal and visual pathways, requiring the integration of object information computed by the ventral stream (form and structure, material properties, surface texture, and functional and conceptual properties) with real-time visuomotor and proprioceptive representations (location of object in egocentric coordinators, the feel and weight of object in hand) supported by parietal computations. Located adjacent to somatosensory region SII and grasp area aIPS (15, 67), the supramarginal gyrus is well placed to integrate proprioceptive and praxis-related information about object grasping and manipulation, with the temporal lobe’s conceptual and goal-related function representations, and the dorsal stream’s real-time visuomotor information about the current layout of peripersonal space. Consequently, processing in the supramarginal gyrus is driven, in part, by inputs from temporal lobe regions that process object identity and conceptual representations of object function (42, 116) (for review and discussion, see ref. 17).

The integration of information across separable processing streams allows for functional grasps to anticipate how the object will be manipulated after it is grasped—a phenomenon known as end-state comfort (117). For instance, if one is to pour water into a glass that is upside on the counter, an awkward initial grasp will be used so that the glass is “right-side up” once it is in-hand. These grasp strategies, crucial in determining an object’s kinematic synergies, rely on interactions among a network of parietal, frontal, and temporal-occipital regions, with several known structural pathways that support parietal to temporal-occipital integration. This includes the vertical occipital fasciculus (118) (connecting posterior lateral occipito-parietal to ventral occipito-temporal regions), the IPS-Fusiform pathways [connecting mid-IPS to mid-fusiform (119), and the vertical arcuate fasciculus, or arcuate temporo-parietal aslant tract (120) (connecting, inter alia, SMG to the middle temporal gyrus). The core proposal here is that the SMG, within a broader network, supports the componential “assembly” of action-relevant representations in ways that are sensitive to, and dependent on, the object’s conceptual and perceptual properties, processed by ventral visual pathway and somatosensory regions, and thus functionally integrating the “what” and “why” of object use with the “how” of action planning.

### Reconciling Two Views of Object Praxis.

Our findings suggest a reconciliation between two theoretical views that have been advanced to explain neuropsychological observations in upper limb apraxia. Patients with upper limb apraxia can have deficits in demonstrating the correct object-associated manipulation, while retaining intact action recognition abilities and conceptual knowledge about the object they fail to correctly use. Importantly, one of the defining features of apraxia, as opposed to motor deficits, is that the disruption to object-directed action is observed for both hands and arms—and not just the contralesional hand/arm. One view (57, 60, 63, 65, 121, 122) has emphasized that an apraxic impairment can arise due to disruptions of stored representations of object-associated manipulations, perhaps analogous to disruption of phoneme or word-level representations in language processing. Another view has argued that object manipulation representations are not stored as precompiled representations but are generated on the fly, de novo, during each specific object interaction (123–127). Our data indicate a critical intermediary step in object-directed actions, supported by the left supramarginal gyrus—the recombination of a closed set of kinematic synergies. This inference is compatible with the view that object-directed manipulations are built on the fly out of kinematic synergies, and with the view that action representations are represented as integrated wholes. By analogy, the existence of word-level representations in language production is not incompatible with their decomposition at a subsequent stage of processing into phonological segments, or further still into subsegmental articulatory features.

Evidence consistent with the presence of both kinematic synergies and integrated action representations is provided by a recent electrocorticography study which investigated common and separable manifolds for finger and grasping movements (78). The authors found that while a common manifold, or covariance structure, characterized a wide range of finger and hand movements across fronto-parietal regions, submanifolds were identified that corresponded to specific movements. Such representations could provide a mechanism to facilitate rapid switching among complex hand movements, even when they are kinematically similar (also see ref. 128). The recipe for how kinematic synergies are combined and weighted may ultimately be what is learned and stored. Large-scale lesion-behavior analyses (56, 129, 130) represent an approach with the power to resolve these matters with causal evidence, and to test how inputs from temporal lobe regions may affect representations of kinematic synergies represented in the supramarginal gyrus.

### Forward Models for Object-Directed Action.

Important questions remain as to the format of kinematic synergies in the left supramarginal gyrus. Our findings, and other related observations (67), indicate that action representations in the left supramarginal gyrus are not reducible to specific motor movements or specific objects, but rather generalize across objects. One possibility, consistent with the available evidence, is that the representations supported by the left supramarginal gyrus are kinesthetic (i.e., somatosensory) rather than *motor* relevant (see refs. 66, 122, 131, and 132 for discussion and evidence). Human SII is directly adjacent to the supramarginal gyrus, as identified herein (133). Furthermore, the types of errors committed by patients with upper limb apraxia may arise due to disrupted representations of the body-in-action. In this regard, a role of the left supramarginal gyrus may be to represent the kinesthetic endpoint, as a target of the action, within a forward model of how a potential object-directed action will unfold (134–136). A forward model of object-directed action must anticipate how actions will feel, given perceptually and conceptually driven inferences about the object properties that will be encountered once the object is touched (such as the surface texture, stiffness of the object, and weight distribution; also see refs. 66, 122, 131, and 132).

Kinematic and perceptual attributes of objects may be integrated, at the granularity of kinematic synergies, by functional connectivity among distal parietal and occipito-temporal regions. That integration, in the context of an action plan or goal, would support kinesthetic and somatosensory representations within a forward model of the action (17, 137, 138). On this view, the dominant supramarginal gyrus supports object-directed action by building a model of each action’s anticipated consequences for the body, in interaction with objects, where the basic building blocks are kinematic synergies.

## Conclusion

A central goal of cognitive brain research is to specify the elemental parts out of which complex higher-order representations are constructed, and to articulate how those parts are functionally and neurally implemented. We have shown that representations of complex-object directed actions in the inferior parietal lobule can be modeled as combinations of finger, hand, wrist, and arm postures and movements—kinematic synergies. These findings motivate a neurocognitive model of how the inferior parietal lobule, in interaction with temporal-occipital and frontal sites, componentially builds complex object-directed actions. In combination with other findings, we suggest that kinematic synergies are combined to form object-directed actions, similarly to how articulatory and voicing features combine to form phonological segments. The role of the inferior parietal lobule in this process may be to support anticipatory representations within a forward model of the kinesthetic and somatosensory consequences of an action for the body. More broadly, and independent of the particular mechanism supported by the supramarginal gyrus, these findings indicate that kinematic synergies are elemental parts that make up object-directed action representations.

## Methods

### Experiment 1: Object Categorization fMRI Experiment.

#### Stimuli.

A set of 80 common manipulable objects and 20 animals (for catch trials) were chosen for this study. There were 800 images of manipulable objects in total, with ten exemplars per manipulable object type. Images were 400-by-400-pixel squares and subtended approximately 10° of the visual angle.

#### Experimental procedure.

Participants (N = 26) completed an event-related fMRI object categorization task and had to identify via button press whether the visually presented object was a tool or an animal catch trial (see *SI Appendix* for detailed participant, experimental procedure, MRI acquisition, and preprocessing). One participant was excluded due to poor data quality prior to any analyses, resulting in a total sample size of N = 25. All experimental procedures were approved by the Ethics Committee of the Faculty of Psychology and Educational Sciences of the University of Coimbra (Portugal) and participants gave written informed consent before testing.

### Experiment 2: Behavioral Synergy Ratings.

#### Stimuli.

A subset of the 80 manipulable objects from Experiment 1 were used in this study, with the addition of the items corkscrew and screwdriver, resulting in a final set of 68 stimuli and three control items (see *SI Appendix* for details).

We selected 54 kinematic hand synergies from the published literature, including 9 basic movements of the wrist, 20 grasp and functional movements (70, 72, 73), and 16 manipulation related movements (71). The same wrist movement synergies were used in the “wrist grasping” and “wrist manipulation” conditions. All synergies were then combined to capture the objects’ kinematic space with 54 synergies × 68 objects. To minimize ambiguity of the movements, a hand was filmed performing each synergy (interacting with a wooden block when necessary) against a green screen. These videos were transformed into gifs that looped (to eliminate memory demands) during stimulus presentation of the object (picture and object label), for the online rating study. Videos are available as part of the data deposition of this study.

#### Experimental procedure.

To obtain ratings of the relevance of each kinematic synergy to each item, Amazon Mechanical Turk (MTurk) participants (N = 87) were asked to indicate which kinematic hand synergies were associated with grasping or manipulating each object in the set of 71 stimuli (68 target objects + three control items). Five fruits were included as catch trials (see *SI Appendix* for details). All experimental procedures were approved by the Carnegie Mellon University Committee on Human Research and participants provided written informed consent before experimental testing.

### Experiment 3: Centrality of Manipulation to Object Function, and Familiarity of Manipulation.

#### Stimuli.

We used the same 71 manipulable object stimuli as in Experiment 2. Just as described above, three of the stimuli served as control items, and the items corkscrew and screwdriver were included to collect ratings but were not present in Experiment 1 (fMRI).

#### Experimental procedure.

We ran a behavioral online experiment (N = 30, mean age = 44.2; see *SI Appendix* for details) with two parts to collect ratings on the centrality of manipulation to object function (part A) and the familiarity to object function (part B). All experimental procedures were approved by the Carnegie Mellon University Committee on Human Research and participants provided written informed consent before experimental testing. In Experiment 3A we asked “How central is the pattern of movement(s) associated with the use of this object/thing in determining its function? (1 = not central; 5 = “very central”).” This locution follows prior work (15) that has shown this dimension effectively separates manipulable objects with a strongly constrained manner of manipulation from those for which there is a variable mapping of manner of manipulation to function. (See *SI Appendix* for the details of Experiment 3B on object function familiarity ratings and testing for performance standards.)

### Experiment 4: fMRI Replication.

#### Stimuli.

The stimuli consisted of nine objects subselected from the stimuli used in Experiment 2 (e.g., bottle opener, clothespin, corkscrew, knife, pencil, pliers, scissors, screwdriver, tweezers). Additionally, six rectangular shapes were included as control stimuli for the participants’ principal task, which was to judge the center of mass of each object (see below).

#### Experimental procedure.

Participants (N = 12) completed a two-session fMRI experiment in which they were presented with the nine object stimuli and asked to indicate, on each trial, the center of mass of that object. All experimental procedures were approved by the Carnegie Mellon University Committee on Human Research and participants provided written informed consent before experimental testing (see *SI Appendix* for detailed participant, experimental procedure, MRI acquisition, and preprocessing).

## Quantification and Statistical Analysis

### Behavioral Synergy Ratings for All Objects (Experiment 2).

We averaged over the subjects’ binary kinematic ratings of the objects, resulting in a group kinematic loading per object, ranging from 0 to 1 (e.g., 54 kinematic features × 68 objects). We then computed a *dissimilarity* matrix (1-r; RDM) among all of the objects that was visualized as a dendrogram using Ward’s criterion. A similarity matrix encoding the similarity among objects based on their kinematic feature correlations—henceforth kinematic representational similarity matrices (kinematic RSM)—was used as an input model for a RSA searchlight, as described below.

### Constructing and Training the Kinematic Encoding Model with Kinematic Synergies.

The predictive encoding model used both the group-averaged behavioral kinematic data of Experiment 2 and the fMRI data from Experiment 1 for the 33 objects judged as having high centrality of manipulation to object function (Experiment 3a). Step 1 of the model computes neural activation maps for each kinematic synergy. For each subject, we created run-level beta maps for each object. Step 1 iterated across each of the 33 objects, each time leaving the to-be-predicted object out of all subsequent steps. For each synergy (for each subject), we selected those objects that had received high (>0.75) and low (<0.25) synergy ratings. For each object in the high group, we subtracted the whole brain map for each of the “low” objects in a pairwise fashion and averaged over the resulting set of subtraction maps to obtain a given synergy’s average whole-brain neural activation map. Repeating this process for all synergies, we obtained an average whole-brain contrast-weighted map for each synergy; critically running the whole process 33 times each time leaving out one object. The left-out-object’s neural activation is predicted in the second step of the model. The object’s (t) predicted activation at every voxel (y_v_) is computed by linearly combining the object’s neural kinematic synergy activations (s_vi_) weighted by their behavioral kinematic rating (k_i_) over all kinematic features (n; see Formula 1). Thus, for each subject, this entire process model was run over 33 folds to obtain a predicted neural activation map for each of the 33 objects.

Experiment 4: In a second, separate analysis, the above steps were repeated using the fMRI data of the 9 manipulable tools from Experiment 4.

### Univariate RSA.

The univariate RSA was designed to measure the covariance between the set of objects’ actual and predicted levels of activation at each voxel.

Experiment1: For each subject in Experiment 1, we generated 33 individual object-specific beta-maps based on the real BOLD fMRI data, and 33 predicted individual object-specific beta-maps. Thus, for each subject, this resulted in two matrices: each containing 33 objects * voxels, one for the real and one for the predicted voxel activations. For each voxel, we then compared the Pearson correlation between the real and the predicted activation (across objects, within a subject). The resulting subject-level r-maps were Fisher z-transformed, normalized to the MNI template, and smoothed with a spatially stationary Gaussian filter (6 mm^3^ full-width-at-half-maximum smoothing kernel). Two-tailed one-sample *t* tests were computed on Fisher z-transformed correlation values to compute univariate group statistics. Threshold-free cluster enhancement (*P* < 0.01) was applied over 10,000 permutations to correct for multiple comparisons and to define statistically significant clusters.

Experiment 3: We computed a univariate RSA for the behavioral centrality and object familiarity ratings from Experiment 3a and 3b. We measured the voxel-level covariance between each objects’ real fMRI activation and the objects’ average behavioral centrality or familiarity/frequency score (collected in independent subjects). The same procedure as above was then applied to the subject-level r-maps before using SPM’s False Discovery Rate (FDR) cluster correction [cluster-forming height-threshold of *P* < 0.001 (139) to correct for multiple comparisons and identify statistically significant clusters of contiguous voxels (two-tailed, *P* < 0.05). Threshold-free cluster enhancement (*P* < 0.01) was applied over 10,000 permutations to correct for multiple comparisons in the object familiarity condition.

Experiment 4: The same procedure was repeated for Experiment 4, with the difference that maps were normalized to Talairach space before identifying statistically significant clusters (two-tailed, *P* < 0.05) via BrainVoyager’s cluster-size statistical threshold estimator and correcting for multiple comparisons with Monte Carlo-style permutation simulations.

## Supplementary Material

Appendix 01 (PDF)

## Data Availability

This study generated video presentations of kinematic synergies (Experiment 2), behavioral kinematic ratings of a large set of manipulable objects (Experiment 2), behavioral judgments of the centrality and familiarity of object use to object function for the set of manipulable objects (Experiment 3), and object-related feature weights of the three CNN models used in the control analyses. Data and materials are available at KiltHub (140). All original code has been deposited at KiltHub and is publicly available as of the date of publication. Any additional information required to reanalyze the data reported in this paper is available from the lead contact upon request.
