# Supplementary material for: Object-directed action representations are componentially built in parietal cortex
Source: Proc Natl Acad Sci U S A. 2025 Aug 20;122(34):e2421032122. doi: 10.1073/pnas.2421032122 (PMC12402996; doi:10.1073/pnas.2421032122)
Supplement: Supplementary file 1 — Appendix 01 (PDF) [file pnas.2421032122.sapp.pdf]

PNAS

Supporting Information for

Object-directed action representations are componentially built in parietal cortex.

Leyla Roksan Caglar, Jon Walbrin, Emefa Akwayena, Jorge Almeida*, & Bradford Z. Mahon*

*Corresponding Authors
Bradford Z. Mahon, Jorge Almeida
Lead Contact
bmahon@andrew.cmu.edu

This PDF file includes:

Supporting text
Figures S1 to S13
Tables S1,S2, and S3
SI References

EXPERIMENTAL MODEL AND STUDY PARTICIPANT DETAILS

Prospective data collection for Experiment 1 was approved by the Ethics Committee of the Faculty of Psychology and Educational Sciences of the University of Coimbra (Portugal). All participants gave written informed consent before testing and received either course credit or monetary compensation for their time. Twenty-six participants (mean age = 25.1, SD = 5.97; 5 male) participated in the fMRI experiment. One participant (subject 10) was excluded from the final analyses due to poor anatomical data quality. All participants were right-handed (self-report) and had normal or corrected to normal vision. Other analyses of the data from Experiment 1, unrelated to the goals of the current report, were reported in Almeida and colleagues (1).

Prospective data collection for Experiments 2, 3, and 4 were approved by the Carnegie Mellon University Committee on Human Research. All participants provided written informed consent before experimental testing. For Experiment 2, a total of 148 participants (mean age = 42.6, SD = 11) were recruited via Amazon Mechanical Turk and completed the Behavioral Kinematics Coding Study on the Qualtrics platform. Participants were assigned to one of four conditions and asked to select all hand movements that were associate with a given manipulable object: movements of the wrist when grasping (N=29), hand synergies when grasping (N=30), movements of the wrist during object manipulation (N=60), and hand movements during object manipulation (N=29). Data quality control checks were interspersed in the online testing, consisting of control items to check task compliance, and a simple fruit vs. tool categorization task (see Experimental Procedures below for details). Sixty-five participants (grasping (N=11), wrist grasping (N=10), manipulation (N=8), wrist manipulation (N=32) did not pass the data quality control checks and were excluded from all subsequent analyses (see Experimental Procedures for details). Eighty-seven participants' data were included in the final analyses for Experiment 2 (mean age = 41.63, SD = 10.86). All participants were native English speakers from the USA, right-handed based on the Edinburgh handedness questionnaire (2) and received monetary compensation for their participation.

For Experiment 3, a total of 30 participants (mean age = 44.2, SD = 10.34**)** were recruited via Amazon Mechanical Turk and completed the Centrality of Manipulation to Object Function, and the Familiarity of Object Use study on the Qualtrics platform after

providing informed consent. All participants were native English speakers from the USA, right-handed based on the Edinburgh handedness questionnaire (Oldfield, 1971) and received monetary compensation for their participation.

For Experiment 4, 12 participants were recruited from Carnegie Mellon University (mean age = 21.92; range: 18-29 years, 9 females, 3 males) to complete a two-session fMRI study. All participants were fluent in English, right-handed, not taking any psychotropic medications, could follow study tasks instructions, and had no history of drug/alcohol abuse, dependence, or traumatic brain injury. Informed consent was obtained from all participants, and upon completion of the experiment, participants were compensated monetarily for their time.

Experiment 1: Object Categorization fMRI Experiment

Experimental procedure

Participants completed an event-related fMRI object categorization task. Each participant completed either 1 (N=26), 2 (N=24), or 3 (N=19) scanning sessions, with three runs in each scanning session. In each run, participants were instructed to maintain fixation on a centered dot and to press one of two buttons (one for each stimulus type) whenever they detected an animal (catch trial) or a tool while being presented with centrally presented images. Assignments of buttons to category were counterbalanced across participants. In a run, participants were presented with one exemplar (presented twice during the run) of each of the 80 manipulable object images for a total of 160 manipulable object trials. Each exemplar was chosen from the 10 possible exemplars per object type. These manipulable object trials were interspaced with 8 animal catch trials (selected from the 20 images of animals), and 54 null events. All trials lasted 4s (the image presented for 2s followed by 2s of fixation; null events were 4s of fixation). Randomization of trial order within each run was applied in the first and second half separately, such that repetitions of stimuli were distributed over the entire run.

MRI data image acquisition and preprocessing

MRI data for Experiment 1 were acquired at the University of Coimbra (BIN – National Brain Imaging Network, Portugal) with a Siemens MAGNETOM Prisma-fit 3T MRI Scanner (Siemens) using a 64-channel head coil. Both functional (T2* weighted (single-shot/GRAPPA) echo-planar imaging pulse sequence, TR = 2000ms, TE = 30ms, flip angle = 75°, 37 interleaved axial slices, acquisition matrix = 70x70 with field of view of 210mm, and voxel size of $3mm^3$) and magnetization prepared rapid gradient echo (MPRAGE) sequence structural T1-weighted images were obtained (TR = 2530 ms, TE = 3.5ms, total acquisition time = 136 s, FA = 7°, acquisition matrix = 256 x 256, with field of view of 256mm, and voxel size of $1mm^3$).

Data were preprocessed in SPM12 applying slice-time correction, realignment, anatomical co-registration, and segmentation. The data was smoothed after normalization to the MNI template (3mm isotropic voxels) only for the univariate RSA analysis (see

below). A general linear model estimation was performed in SPM12 with a high-pass filter of 256s and autoregressive AR(1) model. Run-wise beta maps (1 per condition) were generated along with 6 rigid-motion regressors (and an intercept regressor).

Experiment 2: Behavioral Synergy Ratings

Stimuli

The same 80 manipulable objects from Experiment 1 were used in this study. Eleven of the original 80 objects were excluded for having multiple ways of grasping or manipulating the object. Additionally, we included the items corkscrew and screwdriver (which were not in Experiment 1, but would be included in Experiment 4, see below). This resulted in a final set of 71 stimuli that were normed. Three of those objects (shopping cart, dart, and door handle) were used as control/catch stimuli (see below) and consequently excluded from all analyses, yielding a stimulus set of 68 objects. However, since the items 'corkscrew' and 'screwdriver' were not part of the fMRI dataset, they were not included in the fMRI analyses, resulting in a stimulus set of 66 that overlapped with the items presented during fMRI in Experiment 1.

We selected 54 kinematic hand synergies from the published literature, including 9 basic movements of the wrist, 20 grasp and functional movements (3–5), and 16 manipulation related movements (6). The same wrist movement synergies were used in the "wrist grasping" and "wrist manipulation" conditions. All synergies were then combined to capture the objects' kinematic space (54 synergies x 68 objects). To minimize ambiguity of the movements, a hand was filmed performing each synergy (interacting with a wooden block when necessary) against a green screen. These videos were transformed into gifs that looped (to eliminate memory demands) during stimulus presentation of the object (picture and object label), for the online rating study. Videos are available as part of the data deposition of this study.

Experimental procedure

To obtain ratings of the relevance of each kinematic synergy to each item, Amazon Mechanical Turk (MTurk) participants (N=87) were asked to indicate which kinematic hand synergies were associated with grasping or manipulating each object in the set of 71 items (68 target objects + three control items). Five fruits were included as catch trials.

Participants were instructed to indicate whether the presented synergy is involved in either grasping or manipulating an object in a functionally appropriate manner

(depending on experimental condition). Subjects were instructed what was meant by 'functionally appropriate': Namely, the way in which the object is manipulated when using it according to its intended or designed function or purpose. The experimental conditions consisted of judging the movements of the wrist when grasping, hand synergies when grasping, movements of the wrist during object manipulation, and hand movements during object manipulation. In order to familiarize the participants with the hand movements used in the experiment, they first saw the set of possible hand or wrist synergies in gif format and with the synergy's corresponding label (e.g.: "This is a pinch grasp."). To make it easier to differentiate the different movements, they were presented in two separate groups: movements for which the palm of the hand makes contact with the object and movements for which the palm of the hand does not make contact with the object. Then, in a randomized order, participants saw each one of the 71 objects and the five fruit stimuli with their corresponding labels, once per trial. On each trial, participants had to first indicate whether the object was a fruit or not a fruit and then "whether the palm of the hand makes contact with the object or not" when grasping or manipulating the object to use it according to its function. Depending on the answer, participants were shown the palmar or non-palmar set of synergies and asked to select every kinematic synergy (presented in gif format) that applied to the presented object. This was done to narrow down the synergy choices and limit memory demands, but in an unbiased manner that followed participants' intuitions. Finally, participants were asked to indicate how confident they were with their response (0 = not confident at all; 10 = very confident). Participants that failed to indicate the correct hand movements associated with the three control items or failed the 'fruit attention checks' were excluded from further analyses.

Experiment 3B: Centrality of Familiarity of Manipulation and Estimating Performance Standards

In Experiment 3B, the same participants were asked to rate the familiarity of interacting with each object: "Rate each object based on how frequently you interact with the object/thing with your hands (1 = no experience ever; 7 = daily use)". Prior to providing ratings, participants saw three examples with corresponding answers using the same control stimuli as in Experiment 2: dart, door handle, and shopping cart.

To estimate performance standards, we selected ten items from the dataset (hammer, hairbrush, scissors, magnifying glass, match, board eraser, screw, whistle, syringe, broom), with the intent to represent a range of centrality values. Those items were presented twice during Experiment 3A (Centrality Ratings), allowing us to check the participants' performance consistency. All participants showed high consistency of the repeated items' ratings, measured via a within-subject correlation of the ratings from the first and second presentation: average r (over 30 subjects) = 0.74, SD = 0.27). As an alternative hypothesis, we computed the correlation between the ratings for the first presentation and randomly permuted ratings for the second presentation (1000 permutations). Unlike the high correlation of the real data (r = .74), the distribution of the randomly permuted data showed, as expected, no correlation (r mean = 0.07, SD = 0.13, permutation test vs. real value $p < .0001$), with a range of -0.32 to -0.5. Having confirmed within-subject consistency, only the ratings from the first presentation were used for the final data.

Experiment 4: fMRI Replication

Experimental procedure

Participants completed a two-session fMRI experiment. The first and second session were completed within 14 days of one another. The first session included a 90-minute fMRI scan. While in the scanner, participants were presented with the nine objects and asked to indicate, on each trial, the center of mass of that object. To select their best guess of the balance point (center of mass), participants used a response device to move a vertical line overlaid on the object to the left or right. Pressing down on the response device with the left middle finger moved the slider to the left and pressing with the left index finger moved the slider to the right. All participants practiced the task on a computer and demonstrated proficiency before completing the task in the scanner.

For each trial, objects appeared on the screen for 6 seconds. During this time participants indicated their response by moving the slider marker to the perceived balance point. After the stimulus was presented, a fixation screen was shown for either 8, 10, or 12 seconds, followed by the next 6-second stimulus. Participants completed 4 runs of this task in each scanning session. During each run, participants viewed the 9 manipulable objects in a randomized order and selected the perceived balance point of each. As a control condition, each of these 4 runs included 6 rectangular shaped objects with a blue target line. For those control trials, participants were asked to move the slider to overlap with the target line. That control task served as a baseline on the process of moving the slider to match a target location (for control objects, cued by a line); as the balance task itself is not relevant to the goal of the current study, the control condition was not analyzed further herein. All objects were shown twice in each run (once in their leftward orientation and once in their rightward orientation) resulting in 24 trials for each of the 4 runs of manipulable objects. Although every run consisted of the same 24 objects, a new exemplar of each object was presented in each run to eliminate cross-run stimulus-specific repetition priming effects. Task instruction and stimulus presentation for the second fMRI session were identical to the first session. All data (from all 8 runs per subject) were combined for the analyses presented herein. In the same sessions, participants also completed additional fMRI protocols,

including a category-localizer scan, and additional balance judgement tasks over non-tool stimuli (data not analyzed herein).

MRI data image acquisition and preprocessing

Stimulus presentation and response collection for the Experiment 4 fMRI experiment were coded using PsychoPy 2023.1.3. Data collection took place at the CMU-Pitt Brain Imaging Data Generation & Education (BRIDGE) Center, on a Siemens Prisma 3T MRI scanner, using a 64-channel head coil, and equipped with connectome-level gradients operating at 80mT/m for fMRI testing. Throughout the study, participants laid in a supine position. Functional runs were performed using a gradient echo pulse sequence (TR/TE: 2000/30.00ms, flip angle: 79 degrees, and slick thickness: 2.00 mm and TR/TE: 2300/ 1.99 ms, flip angle: 9 degrees, and slice thickness 1.00 mm, respectively). An anatomical T1-weighted resolution scan (MPRAGE) with the following acquisition parameters was also collected: 128 axial slices per slab, 1.6x 1.6 x 1.6, TE:1.37 ms, TR: 3.15 ms; flip angle = 8 degrees, FOV = 260 mm.

Functional MRI data were analyzed with BrainVoyager 22.2. The analysis pipeline included anatomical preprocessing, functional preprocessing, co-registration of T1 anatomical data with each functional scan, creation of 3D time series files, and a general linear model that included motion as covariates of no interest. Anatomical processing analysis included transformation of the anatomical data into Talairach space. Functional data preprocessing included slice scan time correction (interpolation: cubic spline), 3D motion correction (trilinear/sinc interpolation, voxel resolution inplane X: 2mm, inplane Y: 2mm, slick thickness: 2mm and gap thickness: 0mm), and temporal (high-pass) filtering (2 cycles per run). Spatial smoothing was not applied.

Quantification and Statistical Analysis

Behavioral Function Centrality and Object Familiarity Data (Experiment 3).

For both the centrality ratings (Experiment 3a) and object familiarity ratings (Experiment 3b), we computed a group mean rating by averaging over all subjects' ratings for a given object. Those mean ratings per object were correlated with object-specific BOLD amplitudes in whole-brain univariate analyses.

Based on a ranking of centrality scores, we selected the top 33 objects with the highest scores to construct a subset of objects for which the motor movements were judged central to its function (see Supplemental Figure 1 for distribution of centrality scores). This subset of objects was used for all analyses, unless otherwise noted, of the data from Experiment 1.

Behavioral Similarity Ratings of Manipulable Objects.

Results from a behavioral object piling task, originally reported by Almeida and colleagues (7), were used for adjutant control analyses. Participants were presented with printed names of the objects (to avoid biasing based on image tokens) and asked to think about how similar they are along each of three dimensions: visual appearance, object function, and manner of manipulation. For each dimension, participants were asked to place the objects into piles (by dragging and dropping on a screen) so that the objects within a pile would be similar to each other, but different from objects in other piles (7). A dissimilarity matrix was computed for each dimension based on pile membership of the objects. In our subsequent analyses, we used the dissimilarity matrices for similarity in manner of manipulation (as a comparison to the kinematic state space) and in visual appearance (as one of the visual control RDMs).

Clustering Consistency of the Behavioral Kinematic State Space with Manipulation Feature Clusters.

We compared clustering solutions of the object stimuli, based on the kinematic state space to those based on the explicitly behavioral similarity task [piling task—i.e.: the RDM representing similarity in manner of manipulation from Almeida and colleagues (7). After

computing a dendrogram for both the manipulation RDM and our kinematic space RDM, we calculated their entanglement using R's "dendextend" package (8).

Entanglement quantifies the similarity in hierarchical clustering trees and values range from 0 (perfectly aligned) to 1.0 (completely different). For comparison, a null distribution of entanglement values was then generated by randomly permuting the kinematic dissimilarity matrix 10,000 times, computing its correlation to the manipulation RDM, and fitting a kernel density estimation to the distribution.

Multivariate RSA Searchlight

For each subject in Experiment 1, we used a general linear model (GLM) to obtain run-level beta maps of each object. Representational similarity analysis was run by comparing a model Representational Similarity Matrix (RSM) to neural RSMs using a whole-brain searchlight approach, using Pearson correlation (RSA (9, 10)). The searchlight extraction was run over all objects' beta values to create run-level RSM maps that were then averaged across runs, within each subject. Five different model RSMs were created in total: the behavioral kinematic RSM based on the behavioral kinematic ratings from Experiment 2, and the four visual RSM models (1 behavioral, and 3 CNN models).

All analyses were run in each subject's native space using the CoSMoMVPA package (11) in MATLAB version R2018b before normalizing and registering the subject's searchlight results to MNI space and running group statistics. At the group level, we applied CoSMoMVPA's random-effect Monte Carlo permutation (10,000 iterations) with threshold-free cluster enhancement (TFCE (12)) to correct for multiple comparisons and identify unambiguously statistically significant clusters of contiguous voxels (two-tailed, $p<0.05$).

Visual Feature Models.

Four different models were tested that represented object similarity based on visual features. The first visual RSM was computed using the above-described human behavioral similarity piling task (16). The remaining three visual RSMs were based on pretrained CNNs that were inputted the full stimulus set of Experiment 1. Visual features were

extracted from AlexNet, VGG16, and ResNet-50 [ImageNet 1 K weights from Pytorch (13–15). For AlexNet and VGG16, features were extracted from the maxpool layer after the first convolutional layer (46,656 & 802,816 features, respectively). For ResNet-50 features were extracted from the maxpool layer after the first convolutional layer (200,704 features). The pretrained models were inputted with 10 exemplars for each one of the 80 stimuli. Then, we extracted 10 80×80 representational dissimilarity matrices (RDM; 1-Pearson correlation), assigning one of the 10 exemplars of each target item to each of the RDMs randomly, so that the visual representations would not be biased to a single set of exemplars. Exemplar were Fisher z-transformed and averaged into a final RDM before converting the dissimilarities into similarities (i.e., RDM to RSM), for compatibility with all other analyses.

Region of Interest analysis

A region of interest (ROI) analysis was performed to provide a direct comparison of variance explained by the kinematic encoding model – and the four visual models (high level visual feature model based on behavioral dissimilarities of visual appearance, and three CNNs: AlexNet, ResNet-50, and VGG16). Each model's strength was assessed in 13 anatomically defined ROIs. ROIs were based on the JuBrain SPM Anatomy Toolbox (Version 3.0; (16–18)) and included all parcellations of the inferior parietal cortex (areas PFt, PF, PFm) for the right and left hemisphere separately. ROIs of visual cortex were also included using separate right and left hemisphere areas of Extrastriate cortex (areas hOC3d/hOC4d and hOC3v/hOC4v) as well as combined right and left hemisphere areas of BA 17 and BA 18 (V1/V2). We first calculated the squared Pearson correlation values for each model from each ROI. Then, we computed the contrast in model performance between the kinematic encoding model (KE), to each of the control models (CM), as: [KE-CM]/[KM+CM], and the standard error of the mean. A non-parametric permutation-based ANOVA tested for an interaction between the factors Model and ROI, followed by planned one-sample t-tests to determine if the kinematic encoding model significantly out-performed or underperformed each of the control visual models.

Noise Ceiling Computation

To provide a benchmark for evaluating the kinematic encoding model's performance in the univariate RSA analysis, we computed a noise ceiling to assess the upper bound of inter-subject generalizability. We adopted a pairwise comparison method of every to every other subject. This allowed us to stay closer to the methodology used in the univariate RSA analysis, which aims to measure the covariance between the set of objects' actual and model-predicted levels of activation at each voxel. To obtain a noise ceiling across subjects, we used the individual object-specific beta-maps to assess object-associated agreement between all possible subject pairs. For each voxel, we computed the Pearson correlation across objects, for all possible pairs of subjects, offering an estimation of consistency within the dataset. This noise ceiling estimate served as a reference for interpreting the variance explained by the kinematic encoding models' group-level ROI analyses (see Supplemental Figure 13 and Supplemental Table 3).

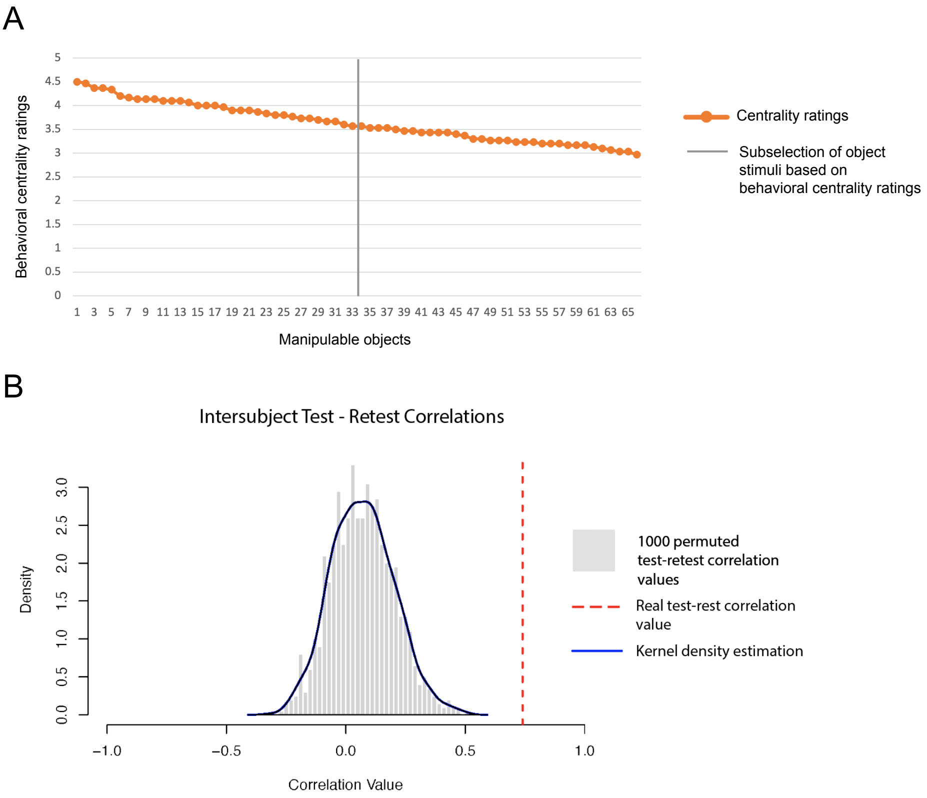
A
Behavioral centrality ratings
Manipulable objects
Centrality ratings
Subselection of object stimuli based on behavioral centrality ratings
B
Intersubject Test - Retest Correlations
Density
Correlation Value
1000 permuted test-retest correlation values
Real test-rest correlation value
Kernel density estimation

Fig. S1. Selection of manipulable object stimuli based on the centrality of an object's manipulation to its function (see Methods, Experiment 3). **A.** Selection of a subset of objects (n=33) was based on choosing the top half of the objects based on the rating of how central the pattern of movement was judged to be to the object's function. The distribution of averaged ratings for the centrality of an object's action to its function is plotted in descending order for all 66 objects. **B.** The empirically measured intersubject test-retest correlation of centrality to object function ratings ($r = 0.74$) is compared to a distribution of 1000 randomly permuted correlation values (range = -0.32 to -0.5, SD = 0.13, mean = 0.07), showing that the real test-retest correlation lies well outside of the distribution of random permutations (permutation test vs. real value $p < .0001$).

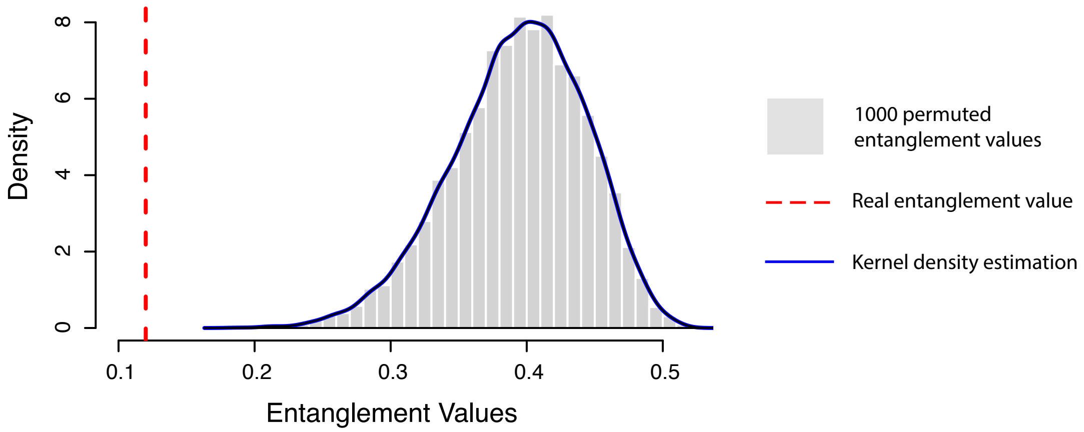

Density
0
2
4
6
8
0.1
0.2
0.3
0.4
0.5
Entanglement Values
1000 permuted entanglement values
Real entanglement value
Kernel density estimation

Fig. S2. The kinematic state space aligns with how humans behaviorally rate object similarity based on associated actions. The alignment between the dendrogram of object similarities based on kinematic synergies (B) and the dendrogram of behaviorally judged manipulation similarity (see Methods) is represented by a low entanglement value of 0.12. For comparison, that measured entanglement value is out of range of a null distribution of entanglement values computed over 10,000 random permutations.

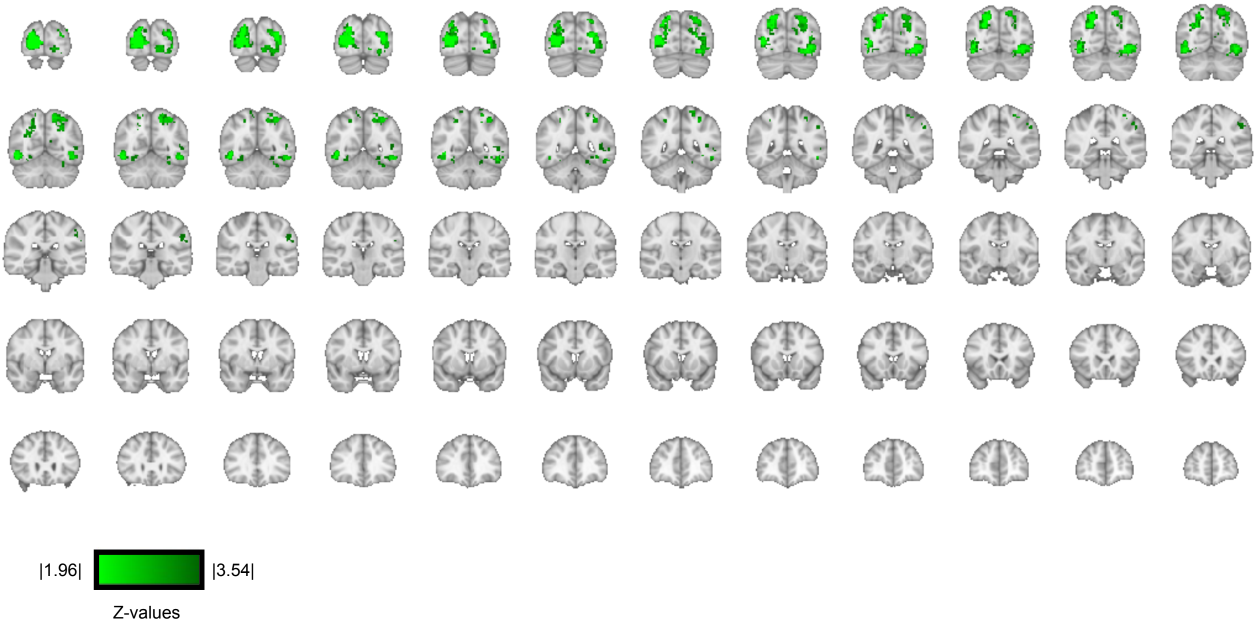
|1.96|
|3.54|
Z-values

Fig. S3. Coronal slices showing that kinematic synergies capture similarity of objects in terms of their associated actions ($p<.05$ TFCE corrected), using a classic multivariate searchlight approach.

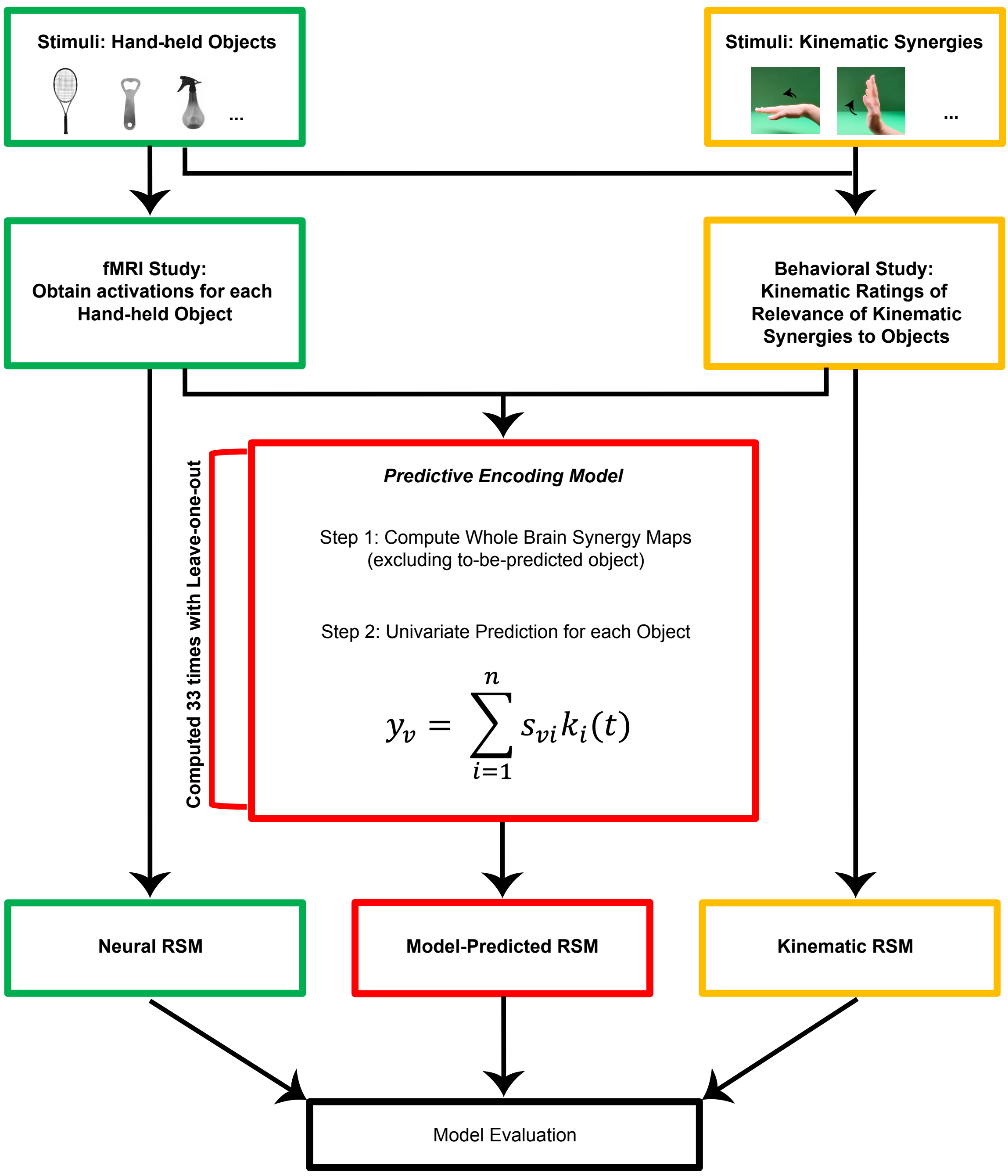

Stimuli: Hand-held Objects
...
Stimuli: Kinematic Synergies
...
fMRI Study:
Obtain activations for each Hand-held Object
Behavioral Study:
Kinematic Ratings of Relevance of Kinematic Synergies to Objects
Computed 33 times with Leave-one-out
Predictive Encoding Model
Step 1: Compute Whole Brain Synergy Maps (excluding to-be-predicted object)
Step 2: Univariate Prediction for each Object
$y_v = \sum_{i=1}^{n} s_{vi} k_i(t)$
Neural RSM
Model-Predicted RSM
Kinematic RSM
Model Evaluation

Fig. S4. Workflow for building and testing the kinematic encoding model. **(Green)** Whole-brain maps for a large set of objects were used to generate neural Representational Similarity Matrices (RSM). **(Yellow)** Naïve participants rated the relevance of each kinematic synergy to the functional use of the objects. The similarity of the objects was computed, as a Kinematic Representational Similarity Matrix (RSM) based on the 54-dimensional kinematic space. **(Green and Yellow)** Unless otherwise noted, analyses were focused on the objects (n = 33) of the broader set (n = 66) that were rated by naïve participants as having the highest centrality of manner of manipulation to object function (Supplemental Figure 1). **(Red)** Whole-brain maps were generated for each kinematic synergy (s_t) from the whole-brain maps of all the objects—leaving the to-be-predicted object out of the entire process of defining the whole-brain synergy maps, through model training, and testing. Univariate whole-brain contrasts subtract the whole brain maps for each object for which the synergy was ranked low (S_L), from the map for objects for which the synergy ranked high (S_H). The kinematic encoding model computes a to-be-predicted object's (t) activation at every voxel (y_v) as the linear combination of the object's neural synergy activations (s_{vi}) weighted by their behavioral kinematic rating (k_i) over all kinematic features (n). In a leave-one-out fashion, the object to be predicted is excluded from all steps and a univariate model-predicted RSM is computed from the model's predicted activation maps: each voxel, for each participant, has a predicted BOLD amplitude for each object. **(Black)** The computational model was evaluated by testing if it predicts neural responses in the theoretically predicted region (i.e.: supramarginal gyrus).

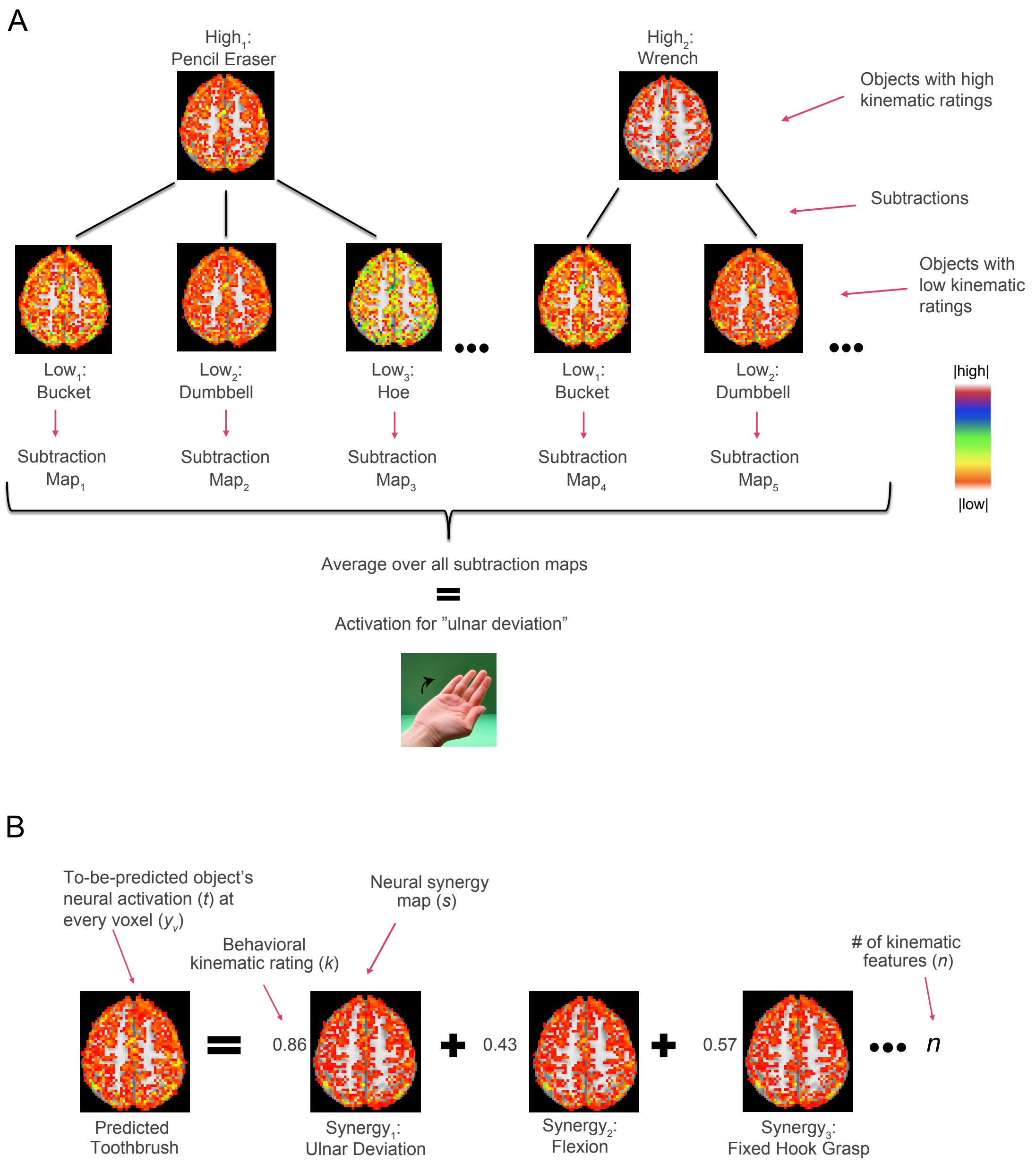

A
High1: Pencil Eraser
High2: Wrench
Objects with high kinematic ratings
Subtractions
Objects with low kinematic ratings
Low1: Bucket
Low2: Dumbbell
Low3: Hoe
Low1: Bucket
Low2: Dumbbell
|high|
|low|
Subtraction Map1
Subtraction Map2
Subtraction Map3
Subtraction Map4
Subtraction Map5
Average over all subtraction maps
=
Activation for "ulnar deviation"
B
To-be-predicted object's neural activation (t) at every voxel (yv)
Neural synergy map (s)
Behavioral kinematic rating (k)
# of kinematic features (n)
= 0.86 + 0.43 + 0.57 ••• n
Predicted Toothbrush
Synergy1: Ulnar Deviation
Synergy2: Flexion
Synergy3: Fixed Hook Grasp

Fig. S5. The kinematic encoding model predicts an object's neural activations based on its associated kinematic synergies, and the learned mapping of kinematic synergies to neural activation for the other objects in the set. **A.** Step 1: A whole-brain activation map was computed for each synergy. For each synergy, the whole-brain maps for objects for which that synergy was rated as being low relevance were iteratively subtracted from the whole-brain maps for objects for which that synergy was rated as being highly relevant. For instance, for the synergy 'ulnar deviation', 'pencil eraser' and 'wrench' were objects for which ulnar deviation was rated as being highly relevant, while 'bucket', 'dumbbell', and 'hoe' were objects for which ulnar deviation was rated as being not relevant. All subtraction maps are averaged to obtain an activation map for the desired synergy. **B.** Step 2: Predicting each object's whole-brain activation map. The model computes a to-be-predicted object's (t) activation at every voxel (y_v) as the linear combination of the object's neural synergy activations (s_{vi}) weighted by their behavioral kinematic rating (k_i) over all kinematic features (n), and the learned mapping from the other objects between kinematic synergies and neural activity.

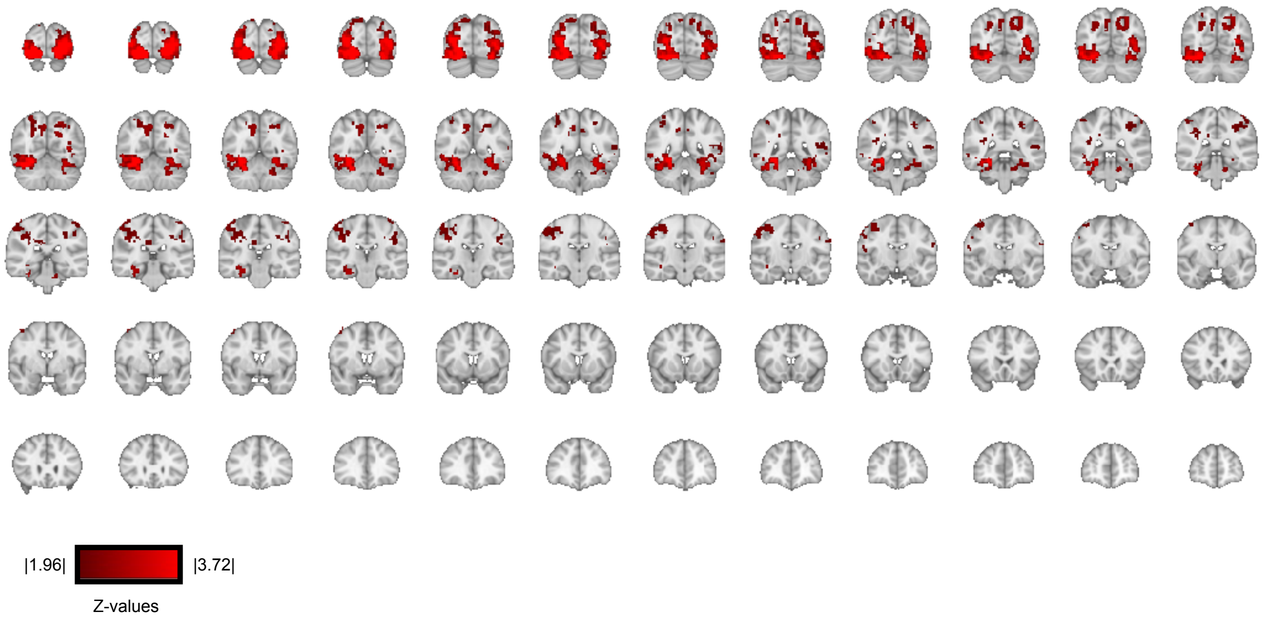
|1.96|
|3.72|
Z-values

Fig. S6. Coronal slices showing the whole-brain prediction of the kinematic encoding model ($p<.05$ TFCE corrected).

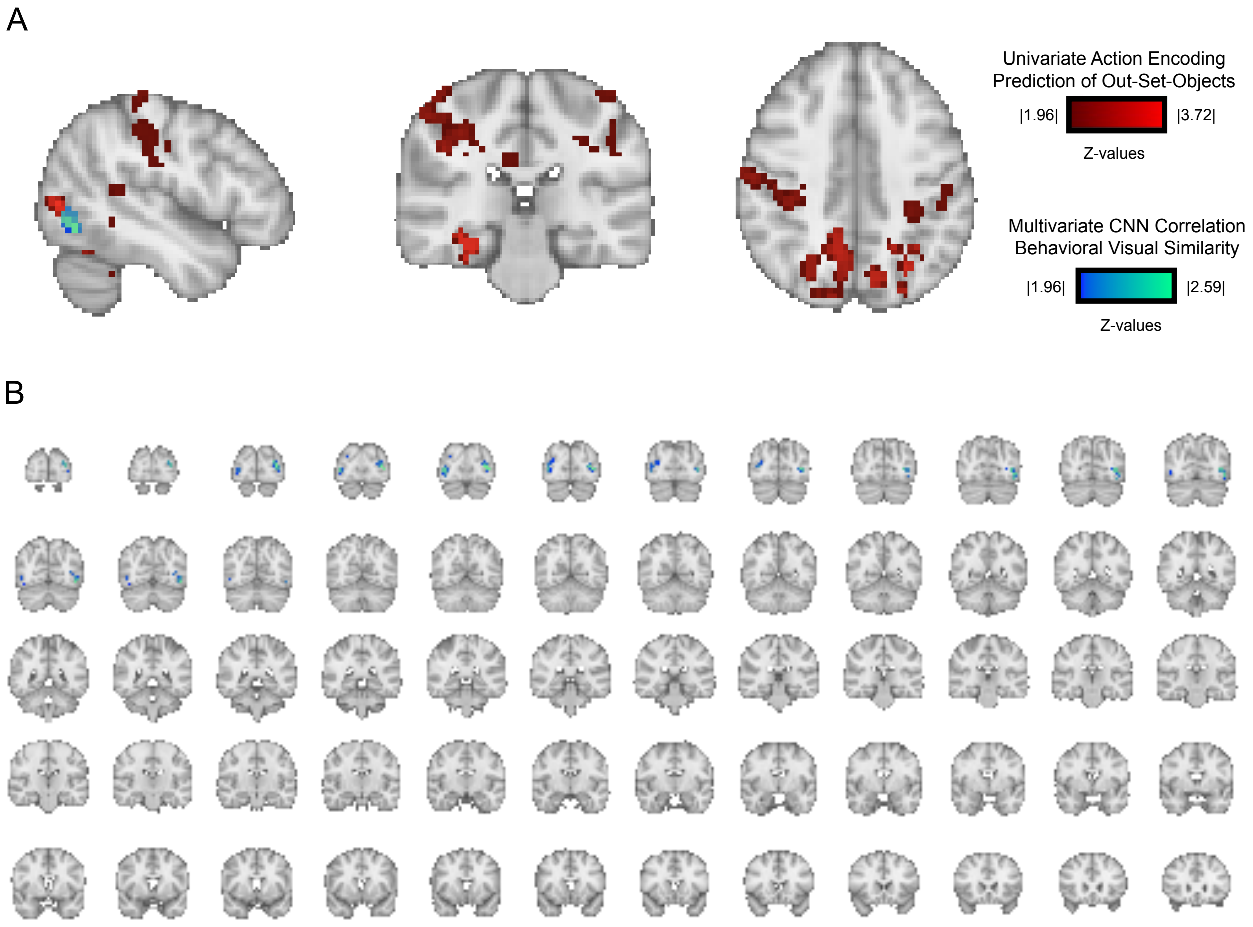

A
Univariate Action Encoding
Prediction of Out-Set-Objects
|1.96|
|3.72|
Z-values
Multivariate CNN Correlation
Behavioral Visual Similarity
|1.96|
|2.59|
Z-values
B

Fig. S7. A. Behavioral judgments of objects' visual structure similarity correlate with neural patterns in lateral occipital cortex but not parietal cortex. The minimum z threshold was set at a liberal 1.96 to demonstrate that even at relaxed thresholds there is no variance explained in the inferior parietal lobule by this measure of visual similarity. **B.** Coronal slices showing the behavioral visual model ($p<.05$ TFCE corrected).

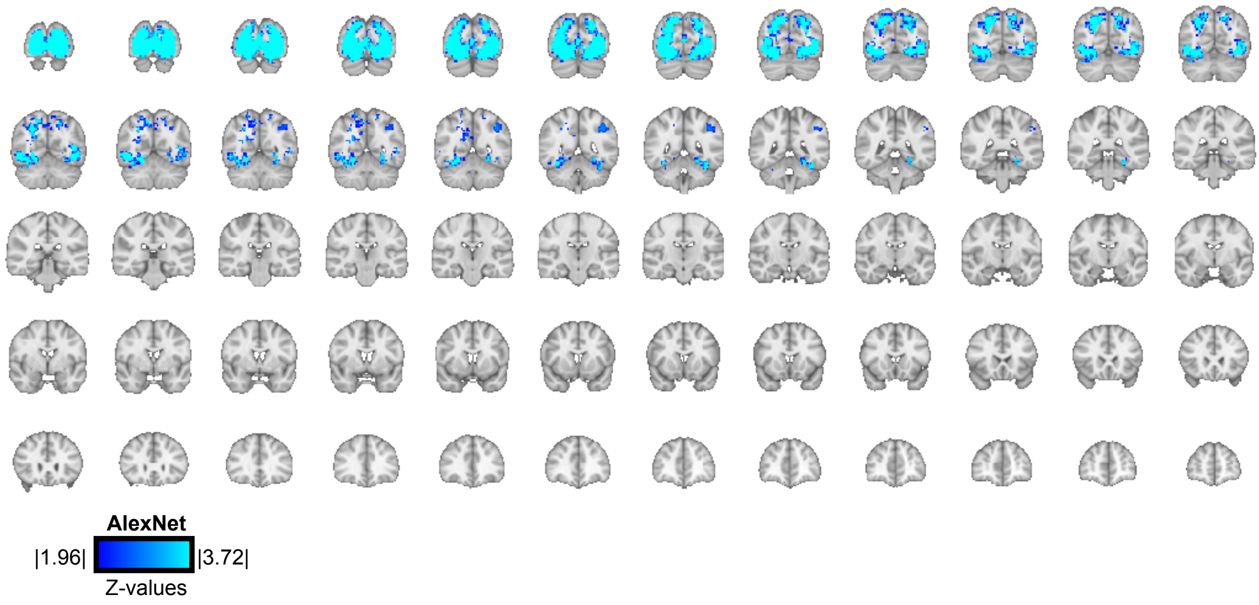
AlexNet
|1.96|
|3.72|
Z-values

Fig. S8. Coronal slices showing that the CNN model AlexNet identifies regions of the ventral and dorsal pathways but does not identify the supramarginal gyrus ($p<.05$ TFCE corrected).

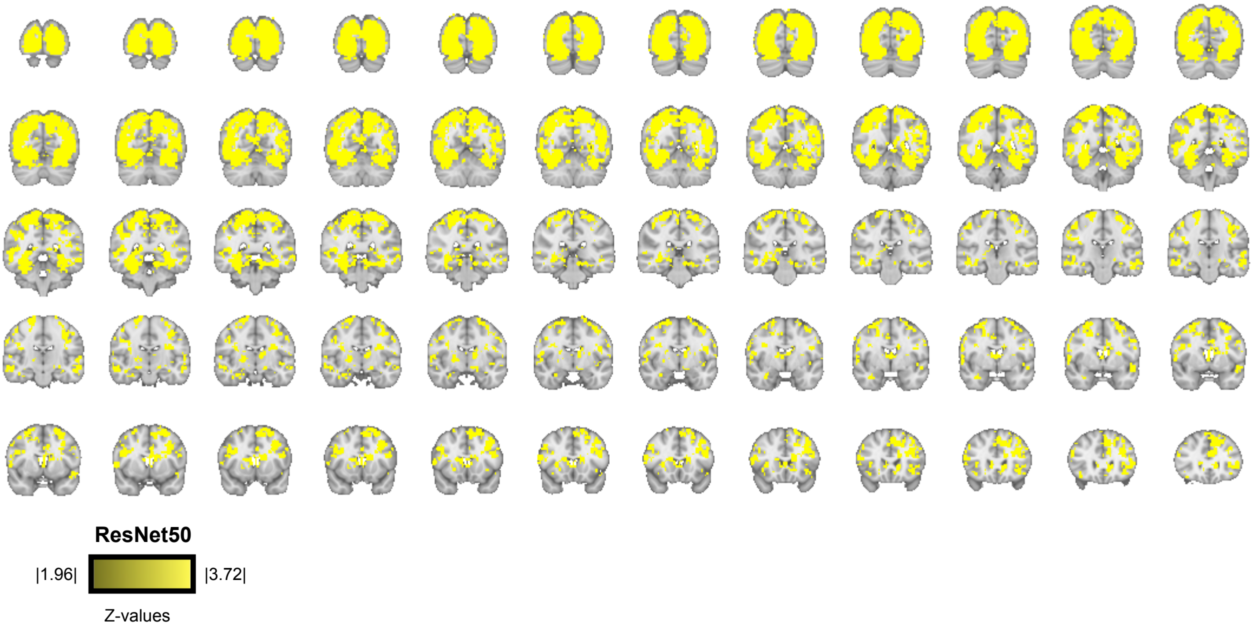

ResNet50
|1.96|
|3.72|
Z-values

Fig. S9. Coronal slices showing the CNN model ResNet50 identifies regions of the ventral and dorsal pathways but does not identify the supramarginal gyrus ($p<.05$ TFCE corrected).

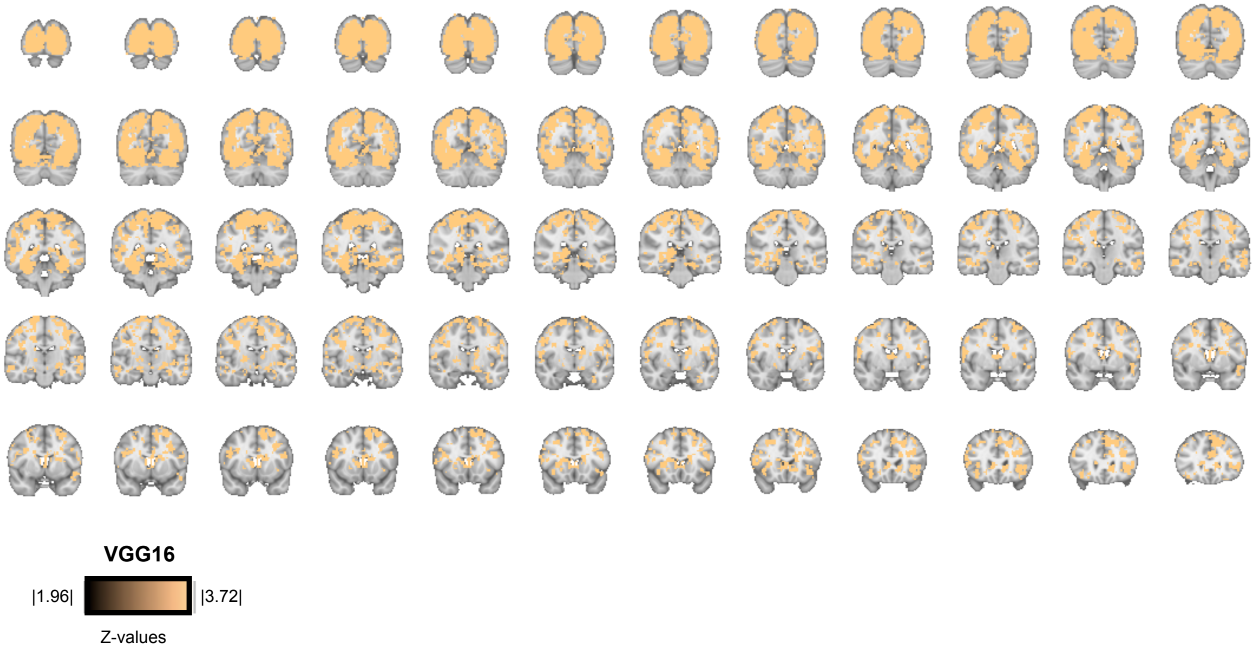
VGG16
|1.96|
|3.72|
Z-values

Fig. S10. Coronal slices showing the CNN model VGG16 identifies regions of the ventral and dorsal pathways but does not identify the supramarginal gyrus ($p<.05$ TFCE corrected).

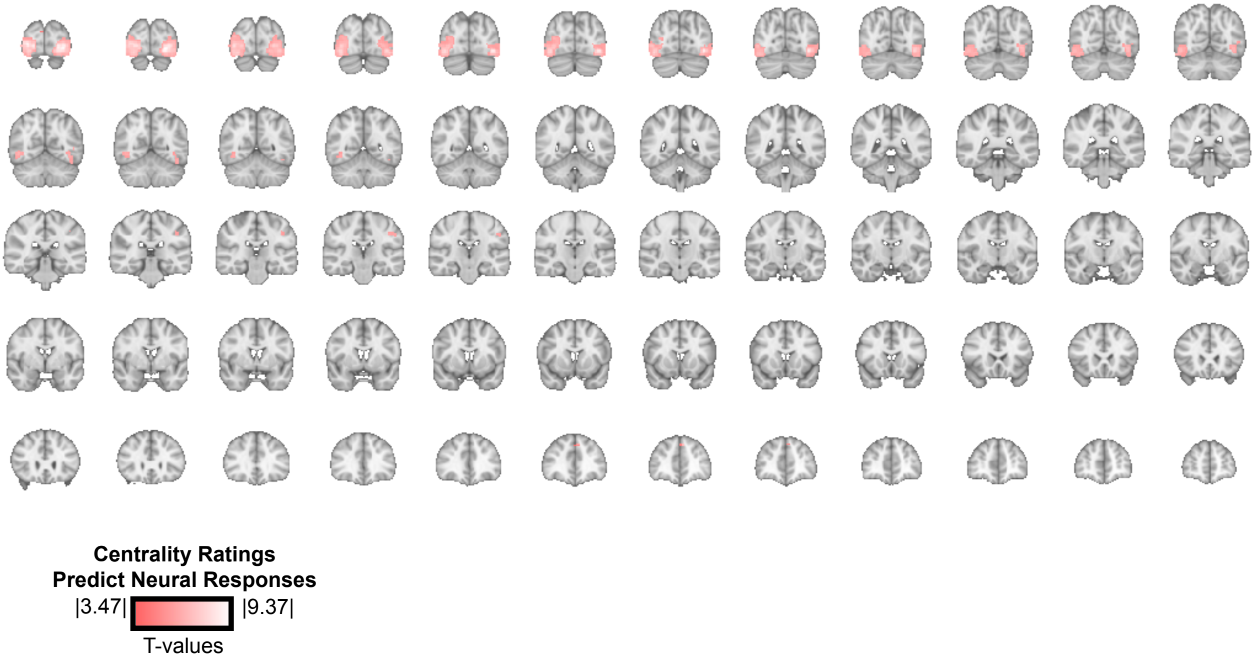

Centrality Ratings
Predict Neural Responses
|3.47|
|9.37|
T-values

Fig. S11. Coronal slices of voxel-wise correlation between fMRI BOLD amplitude and centrality ratings. This shows that the centrality of each object's action to its function predicts neural responses in the supramarginal gyrus ($p<.05$ FDR corrected).

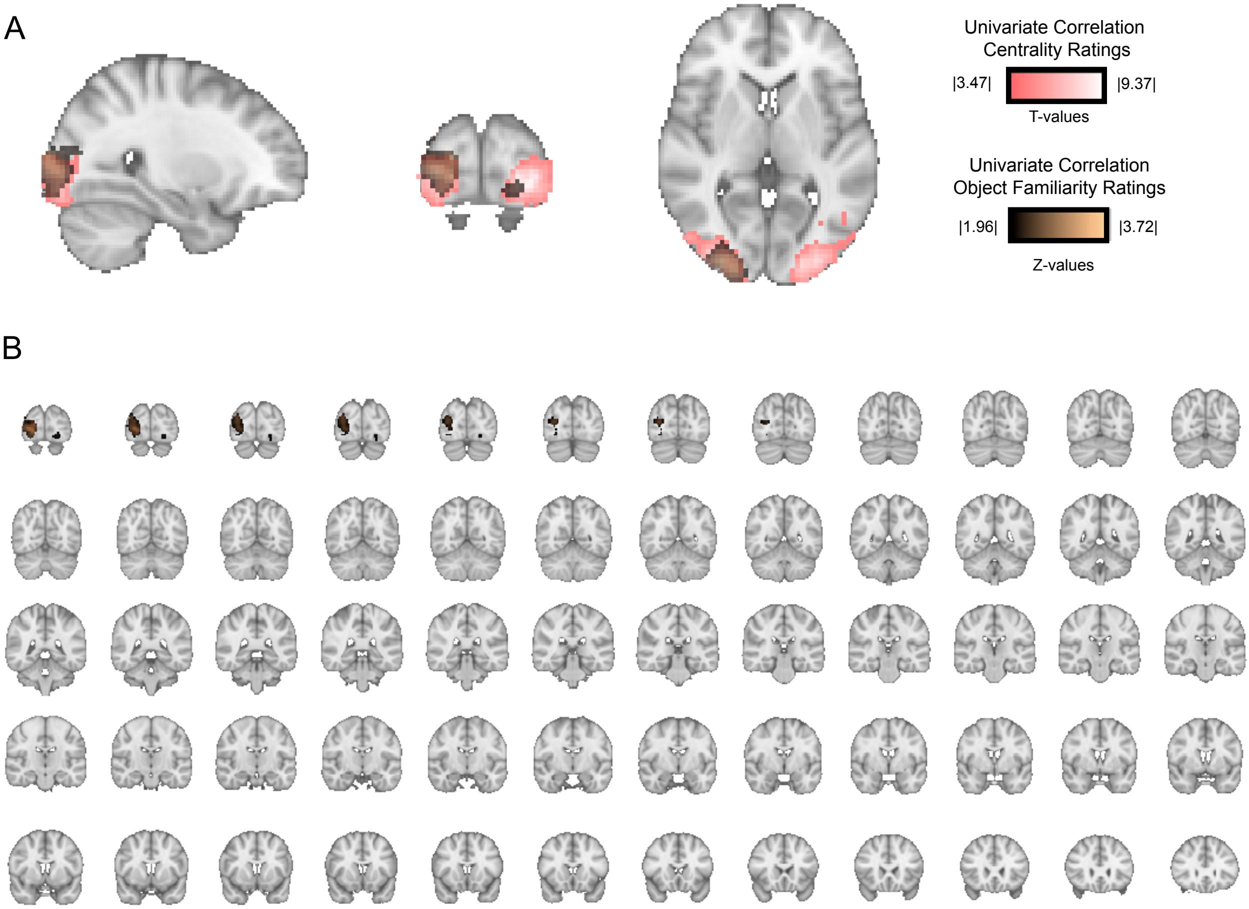

A
Univariate Correlation
Centrality Ratings
|3.47|
|9.37|
T-values
Univariate Correlation
Object Familiarity Ratings
|1.96|
|3.72|
Z-values
B

Fig. S12. Control analysis shows that the familiarity of object use is not coded in the supramarginal gyrus. **A.** For context, centrality of an object's action to its function is shown again (pink). The familiarity of object use (brown) does not code for activity in the supramarginal gyrus but does identify visual areas ($p<.05$ TFCE corrected). **B.** Coronal slices of voxel-wise correlation between fMRI BOLD amplitude and object familiarity ratings**.**

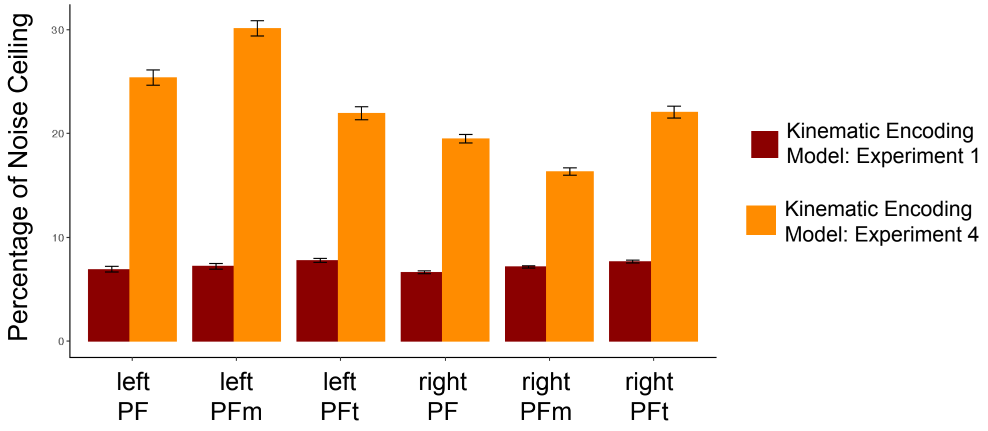
Percentage of Noise Ceiling
30
20
10
0
left PF
left PFm
left PFt
right PF
right PFm
right PFt
Kinematic Encoding Model: Experiment 1
Kinematic Encoding Model: Experiment 4

Fig. S13. The graph plots the kinematic encoding models from Experiments 1 and 4 (r values), as a percent of an estimated noise ceiling ([Model/Noise Ceiling Estimate]*100). The graph shows that the steps taken to optimize signal-to-noise (item selection and a temporally slower fMRI design) in the replication (Experiment 4) afforded the kinematic encoding model to explain a substantially higher amount of variance in the inferior parietal lobule as compared to Experiment 1.

Table S1. The anatomical location of the core findings herein, showing that responses in the inferior parietal lobule can be modeled as linear combinations of kinematic synergies, are anatomically well aligned based on coordinates identified in the literature. The studies below were chosen for using ‘tool’ stimuli selected as having high Centrality of Manipulation to function. Note that some studies do not distinguish SMG (supramarginal gyrus) from aIPS (anterior intraparietal sulcus).

**Study**	**ROIs**	**Talairach coordinates**		
		**x**	**y**	**z**
Almeida et al. (1)	Inferior left parietal cortex	-33	-34	28
Chao & Martin (14)	L posterior parietal (viewing task)	-32 +/-5	-47 +/-9	42 +/-6
	L posterior parietal (naming task)	-30 +/-3	-39 +/-2	47 +/-1
Chen, Garcea, Almeida, & Mahon (15)	R hand area	-33 +/-4.3	-29 +/-4.5	52 +/-3.2
	L aIPS tool area	-36 +/-6.4	-47 +/-7.7	43 +/-6.9
Chen, Garcea, Almeida, & Mahon (16)	L inferior parietal lobule	-34 +/-7.8	-46 +/-9.0	47 +/-7.5
Chen, Garcea, Jacobs, & Mahon (17)	L Inferior parietal lobule (SMG/aIPS): pantomime task	-45	-34	42
	L Inferior parietal lobule (SMG/aIPS): identification task	-60	-37	25
	L Inferior parietal lobule (SMG/aIPS): cross-task: pantomime to identification	-57	-28	28
	L Inferior parietal lobule (SMG/aIPS): cross-task: identification to pantomime	-48	-13	16
Mahon et al. (18)	L inferior parietal lobule (SMG)	-57	-27	34
		**MNI coordinates**		
Chen, Snow, Culham, & Goodale (19)	L aIPS (tools > nontools)	-36	-43	52
	R aIPS (tools > nontools)	27	-43	46
	L aIPS and aSMG (tools > animals)	-48	-32	41

Table S2. P-values, adjusted p-values, and Cohen's d values for the ROI analysis presented in Figure 3C. All values were computed based on the model performance contrast between the visual models and the kinematic encoding model of Experiment 1.

	**Kinematic Encoding vs. AlexNet**			**Kinematic Encoding vs. ResNet50**			**Kinematic Encoding vs. VGG16**		
**ROI**	**P-value**	**Adj. P-value**	**Cohen's d**	**P-value**	**Adj. P-value**	**Cohen's d**	**P-value**	**Adj. P-value**	**Cohen's d**
rhOc3d	9.00E-08	3.50E-06	-1.51	1.50E-01	1.00E+00	-0.29	7.60E-01	1.00E+00	-0.06
lhOc3v	3.00E-01	1.00E+00	-0.21	1.90E-04	7.30E-03	0.88	1.30E-06	5.10E-05	1.28
lhOc4d	1.80E-03	6.90E-02	-0.7	5.10E-02	1.00E+00	0.41	7.50E-03	2.90E-01	0.58
rhOc3d	1.00E-04	4.00E-03	-0.93	3.20E-01	1.00E+00	-0.21	9.30E-01	1.00E+00	-0.02
rhOc3v	7.90E-02	1.00E+00	-0.37	3.20E-06	1.30E-04	1.2	4.30E-08	1.70E-06	1.57
rhOc4d	9.70E-06	3.80E-04	-1.12	7.20E-01	1.00E+00	0.07	4.90E-01	1.00E+00	0.14
V1 & V2	2.80E-02	1.00E+00	-0.47	3.40E-02	1.00E+00	0.45	1.10E-03	4.20E-02	0.74
lPF	8.50E-20	3.30E-18	-5.58	6.30E-16	2.40E-14	-3.78	1.20E-13	4.70E-12	-2.99
lPFm	8.70E-18	3.40E-16	-4.57	1.20E-13	4.90E-12	-2.98	1.00E-12	3.90E-11	-2.71
lPFt	1.20E-11	4.60E-10	-2.41	1.60E-04	6.40E-03	-0.89	3.50E-03	1.30E-01	-0.65
rPF	7.90E-19	3.10E-17	-5.07	1.10E-12	4.10E-11	-2.7	5.20E-11	2.00E-09	-2.24
rPFm	3.10E-15	1.20E-13	-3.53	1.30E-07	5.20E-06	-1.47	5.00E-07	2.00E-05	-1.36
rPFt	1.30E-03	5.00E-02	-0.73	6.80E-01	1.00E+00	-0.08	8.00E-01	1.00E+00	0.05

Table S3. Variance explained by the kinematic encoding models in each parietal cortex ROI. For each model and ROI, we report the region's average correlation value, variance explained based on r-squared values, and the standard error of the mean (SEM) on the correlation values. This is shown for all individual ROIs of the encoding model data of Experiments 1 and 4.

	**Experiment 1**			**Experiment 4**		
**ROIs**	**r**	**r2**	**SEM on r**	**r**	**r2**	**SEM on r**
**lPF**	0.0339	0.0011	0.0005	0.1240	0.0154	0.0036
**lPFm**	0.0311	0.0010	0.0005	0.1299	0.0169	0.0031
**lPFt**	0.0427	0.0018	0.0006	0.1208	0.0146	0.0034
**rPF**	0.0406	0.0017	0.0004	0.1196	0.0143	0.0025
**rPFm**	0.0441	0.0019	0.0005	0.1008	0.0102	0.0022
**rPFt**	0.0438	0.0019	0.0006	0.1261	0.0159	0.0033
**Averages**	**0.039**	**0.002**	**0.001**	**0.120**	**0.015**	**0.003**

References

1. J. Almeida, A. R. Fintzi, B. Z. Mahon, Tool manipulation knowledge is retrieved by way of the ventral visual object processing pathway. *Cortex* **49**, 2334–2344 (2013).

2. R. C. Oldfield, The assessment and analysis of handedness: the Edinburgh inventory. *Neuropsychologia* **9**, 97–113 (1971).

3. M. Atzori, *et al.*, Electromyography data for non-invasive naturally-controlled robotic hand prostheses. *Sci. Data* **1**, 1–13 (2014).

4. N. J. Jarque-Bou, M. Atzori, H. Müller, A large calibrated database of hand movements and grasps kinematics. *Sci. Data* **7**, 1–10 (2020).

5. F. Stival, *et al.*, A quantitative taxonomy of human hand grasps. *J. Neuroengineering Rehabil.* **16**, 1–17 (2019).

6. I. M. Bullock, A. M. Dollar, Classifying human manipulation behavior in *2011 IEEE International Conference on Rehabilitation Robotics*, (IEEE, 2011), pp. 1–6.

7. J. Almeida, *et al.*, Neural and behavioral signatures of the multidimensionality of manipulable object processing. *Commun. Biol.* **6**, 940 (2023).

8. T. Galili, dendextend: an R package for visualizing, adjusting and comparing trees of hierarchical clustering. *Bioinformatics* **31**, 3718–3720 (2015).

9. N. Kriegeskorte, M. Mur, P. A. Bandettini, Representational similarity analysis-connecting the branches of systems neuroscience. *Front. Syst. Neurosci.* **4** (2008).

10. H. Nili, *et al.*, A toolbox for representational similarity analysis. *PLoS Comput. Biol.* **10**, e1003553 (2014).

11. N. N. Oosterhof, A. C. Connolly, J. V. Haxby, CoSMoMVPA: multi-modal multivariate pattern analysis of neuroimaging data in Matlab / GNU Octave. *Front. Neuroinformatics* (2016). https://doi.org/10.3389/fninf.2016.00027.

12. S. M. Smith, T. E. Nichols, Threshold-free cluster enhancement: addressing problems of smoothing, threshold dependence and localisation in cluster inference. *Neuroimage* **44**, 83–98 (2009).

13. A. Krizhevsky, I. Sutskever, G. E. Hinton, ImageNet Classification with Deep Convolutional Neural Networks in *Advances in Neural Information Processing Systems*, (Curran Associates, Inc., 2012).

14. K. He, X. Zhang, S. Ren, J. Sun, Deep residual learning for image recognition in *Proceedings of the IEEE Conference on Computer Vision and Pattern Recognition*, (2016), pp. 770–778.

15. K. Simonyan, A. Zisserman, Very deep convolutional networks for large-scale image recognition. (2014).

16. S. B. Eickhoff, *et al.*, A new SPM toolbox for combining probabilistic cytoarchitectonic maps and functional imaging data. *Neuroimage* **25**, 1325–1335 (2005).

17. S. B. Eickhoff, S. Heim, K. Zilles, K. Amunts, Testing anatomically specified hypotheses in functional imaging using cytoarchitectonic maps. *Neuroimage* **32**, 570–582 (2006).

18. S. B. Eickhoff, *et al.*, Assignment of functional activations to probabilistic cytoarchitectonic areas revisited. *Neuroimage* **36**, 511–521 (2007).

19. L. L. Chao, A. Martin, Representation of manipulable man-made objects in the dorsal stream. *Neuroimage* **12**, 478–484 (2000).

20. Q. Chen, F. E. Garcea, B. Z. Mahon, The representation of object-directed action and function knowledge in the human brain. *Cereb. Cortex* **26**, 1609–1618 (2016).

21. Q. Chen, F. E. Garcea, J. Almeida, B. Z. Mahon, Connectivity-based constraints on category-specificity in the ventral object processing pathway. *Neuropsychologia* **105**, 184–196 (2017).

22. Q. Chen, F. E. Garcea, R. A. Jacobs, B. Z. Mahon, Abstract representations of object-directed action in the left inferior parietal lobule. *Cereb. Cortex* **28**, 2162–2174 (2018).

23. B. Z. Mahon, *et al.*, Action-related properties shape object representations in the ventral stream. *Neuron* **55**, 507–520 (2007).

24. J. Chen, J. C. Snow, J. C. Culham, M. A. Goodale, What role does "elongation" play in "tool-specific" activation and connectivity in the dorsal and ventral visual streams? *Cereb. Cortex* **28**, 1117–1131 (2018).
